# Real‐world progression‐free survival and overall survival in patients with HR ^+^/HER2 ^−^ advanced breast cancer treated in first‐line with ribociclib, endocrine monotherapy or chemotherapy: Results from the observational RIBANNA study

**DOI:** 10.1002/ijc.70397

**Published:** 2026-03-06

**Authors:** Peter A. Fasching, Cosima Brucker, Thomas Decker, Anne Engel, Thomas Göhler, Christian Jackisch, Jan Janssen, Andreas Köhler, Kerstin Lüdtke‐Heckenkamp, Diana Lüftner, Frederik Marmé, Marion van Mackelenbergh, Beate Rautenberg, Marcus Schmidt, Rudolf Weide, Pauline Wimberger, Elena Kisseleff, Christina Pfister, Claudia Quiering, Christian Roos, Achim Wöckel

**Affiliations:** ^1^ Department of Obstetrics and Gynecology University Hospital Erlangen Erlangen Germany; ^2^ Department of Obstetrics and Gynecology Klinikum Nürnberg Nord, Paracelsus Medical University Nürnberg Germany; ^3^ Oncology Ravensburg Ravensburg Germany; ^4^ Winicker Norimed GmbH Nürnberg Germany; ^5^ Onkozentrum Dresden/Freiberg Dresden Germany; ^6^ Evangelische Kliniken Essen Mitte gGmbH (KEM) Essen Germany; ^7^ Medizinische Studiengesellschaft Nord‐West GmbH Westerstede Germany; ^8^ Practice for Hematology and Oncology Langen Germany; ^9^ Department of Oncology and Hematology Niels‐Stensen Kliniken Georgsmarienhütte Germany; ^10^ Immanuel Hospital Märkische Schweiz, Buckow & Immanuel Campus Rüdersdorf, Medical University of Brandenburg Theodor Fontane Rüdersdorf bei Berlin Germany; ^11^ Department of Gynecology and Obstetrics Medical Faculty Mannheim, Heidelberg University Mannheim Germany; ^12^ Department of Gynecology and Obstetrics University Hospital Schleswig‐Holstein Kiel Germany; ^13^ Medical Center–University of Freiburg Freiburg Germany; ^14^ Department of Gynecology and Obstetrics University Medical Center of the Johannes Gutenberg University Mainz Mainz Germany; ^15^ Institut für Versorgungsforschung in der Onkologie Koblenz Germany; ^16^ Department of Gynecology and Obstetrics University Hospital Carl Gustav Carus, Technische Universität Dresden, and NCT Dresden Dresden Germany; ^17^ Novartis Pharma GmbH Nürnberg Germany; ^18^ Department of Obstetrics and Gynecology University Hospital Würzburg Würzburg Germany

**Keywords:** CDK4/6 inhibitors, metastatic breast cancer, observational study, overall survival, ribociclib

## Abstract

Cyclin‐dependent kinase 4 and 6 inhibitors (CDK4/6i) combined with endocrine therapy are the preferred choice for first‐line treatment of patients with HR^+^/HER2^−^ locally advanced/metastatic breast cancer (aBC). The CDK4/6i ribociclib in combination with an aromatase inhibitor (AI) or fulvestrant (FUL) has demonstrated significant progression‐free survival (PFS) and overall survival (OS) benefits for pre‐ and postmenopausal aBC patients who were enrolled in the three pivotal MONALEESA trials. Following the initial approval of ribociclib in 2017, the non‐interventional RIBANNA study was initiated to evaluate the effectiveness and safety of ribociclib plus AI/FUL therapy among patients with aBC in a real‐world setting. Two additional treatment cohorts (endocrine monotherapy [ET] and chemotherapy [CT]) were included to extend the knowledge about current aBC treatments. A total of 2567 patients were enrolled in 279 study centers, of whom 1852 were treated with ribociclib+AI/FUL, 183 were treated with ET, and 139 were treated with CT, who were available for effectiveness analyses. Median PFS (mPFS) and median OS (mOS) on first‐line treatment with ribociclib+AI/FUL were 35.0 and 76.0 months, respectively. Adjustment for differences in demographic and baseline characteristics resulted in a longer mPFS on ribociclib+AI/FUL (34.7 months) compared to ET (26.4 months) or CT (19.2 months). Adverse events (AEs) on ribociclib were consistent with those seen in the pivotal trials, and no new safety signals were observed. The RIBANNA study confirmed the PFS and OS benefit seen in the MONALEESA trials. Together with the safety data, this large real‐world dataset supports the favorable risk/benefit profile of ribociclib in large scale patient populations.

## What's New?

The CDK4/6 inhibitor ribociclib in combination with an endocrine‐based therapy has demonstrated significant progression‐free and overall survival benefits in patients with advanced/metastatic HR+/HER2‐ breast cancer in the pivotal MONALEESA trials. This large prospective, observational RIBANNA study corroborates the survival benefits and safety profile of ribociclib in routine clinical practice. The findings support ribociclib plus endocrine therapy as first‐line treatment for HR+/HER2‐ advanced/metastatic breast cancer. The RIBANNA study confirms that ribociclib unfolds its beneficial effects in a real‐world setting, even in patients who were not covered by the pivotal trials.

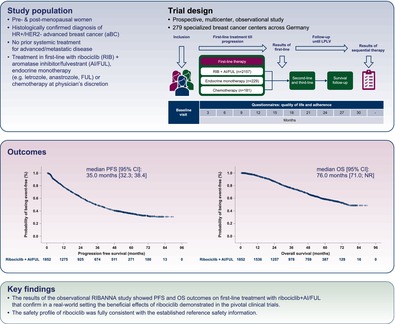

**Abbreviations**
aBC: advanced/metastatic breast cancer
AE: adverse event
AGO: Arbeitsgemeinschaft Gynäkologische Onkologie (German Gynecological Oncology Group)
AI: aromatase inhibitor
BC: breast cancer
BfArM: Bundesinstitut für Arzneimittel und Medizinprodukte (Federal Institute for Drugs and Medical Devices)
CDK: cyclin‐dependent kinase
CDK4/6i: cyclin‐dependent kinase 4/6 inhibitor
CI: confidence interval
CNS: central nervous system
CT: chemotherapy
CTCAE: common terminology criteria for adverse events
DFI: disease‐free interval
ECOG: Eastern Cooperative Oncology Group
eCRF: electronic case report form
ENR: enrolled patient population
EORTC: European Organization for Research and Treatment of Cancer
ESMO: European Society for Medical Oncology
ET: endocrine therapy
EU: European Union
FAS: full analysis set
HER2: human epidermal growth factor receptor 2
HR: hazard ratio
HR+: hormone receptor‐positive
IC: informed consent
ISPE: International Society for Pharmaceutical Engineering
KM: Kaplan‐Meier
MCBS: Magnitude of Clinical Benefit Scale (ESMO)
MedDRA: medical dictionary for regulatory activities
ML: MONALEESA
mOS: median overall survival
mPFS: median progression‐free survival
NCCN: National Comprehensive Cancer Network
NIS: non‐interventional study
NR: not reached
NSAI: non‐steroidal aromatase inhibitor
OS: overall survival
PEI: Paul Ehrlich Institute
PFS: progression‐free survival
PS: performance status
SAE: serious adverse event
SAF: safety analysis set
SmPC: summary of product characteristics
STROBE: strengthening the reporting of observational studies in epidemiology
US: United States (of America)
WHO: World Health Organization

## 1 INTRODUCTION

Among women, breast cancer is the most common malignant disease worldwide, accounting for 24% of new cancer cases and 15% of cancer deaths in 2018, and incident cases are expected to increase by more than 46% by 2040.^1^ In 2022, 2.3 million women were diagnosed with breast cancer and 670,000 related deaths were registered worldwide according to WHO data,^2^ while 374,800 new cases and 95,800 deaths were estimated for the European Union.^3^ In Germany, 74,512 new cases and more than 18,512 deaths were estimated for 2022.^4^ Thus, breast cancer remains a major burden for affected patients and health care systems.

The receptor expression pattern plays an important role for the choice of therapy for locally advanced/metastatic breast cancer (aBC). About 70% of breast cancer patients show a hormone receptor‐positive (HR^+^) and human epidermal growth factor receptor 2‐negative (HER2^−^) receptor status.^5^ Current guidelines recommend first‐line therapy with a CDK4/6i in combination with endocrine therapy (ET) for patients with HR^+^/HER2^−^ aBC.^6^, ^7^, ^8^, ^9^, ^10^ Currently chemotherapy for HR^+^/HER2^−^ aBC is only indicated in exceptional cases and must be well justified as an individual decision.^11^

CDK4/6i primarily act by targeted and selective inhibition of the CDK4/6 complex, thereby leading to downstream modulation of cell cycle progression.^12^ The three currently approved CDK4/6i (palbociclib, abemaciclib, and ribociclib) combined with ET have demonstrated their superiority in prolonging progression‐free survival (PFS) compared with ET alone in pre‐ or postmenopausal women with HR^+^/HER2^−^ aBC.^13^, ^14^, ^15^, ^16^, ^17^, ^18^, ^19^ The clinical effects of ribociclib have been extensively studied in the three pivotal, placebo‐controlled, Phase 3 trials MONALEESA‐2^20^, ^21^ (first‐line in combination with letrozole; post‐menopausal), MONALEESA‐3^17^, ^22^, ^23^, ^24^ (first‐ or second‐line in combination with fulvestrant; post‐menopausal), and MONALEESA‐7^19^, ^25^ (mostly first‐line in combination with NSAI or tamoxifen; pre‐menopausal).

Ribociclib has shown statistically significant and clinically meaningful PFS and overall survival (OS) benefit consistently in all three Phase 3 MONALEESA randomized clinical trials and is the only CDK4/6i to demonstrate significant OS benefit across all first‐line Phase 3 aBC trials.^17^, ^19^, ^20^, ^21^, ^24^, ^25^, ^26^, ^27^, ^28^ Current national AGO guidelines recommend the preferred use of ribociclib for pre‐ and post‐menopausal standard therapy in aBC, if combined with an NSAI or fulvestrant, and additionally GnRH analogs in case of premenopausal status, even for clinically aggressive disease (AGO++^29^). ESMO guidelines assign ribociclib+ET the highest MCBS score of 4 (substantial) amongst all CDK4/6i for first‐line treatment of HR^+^/HER2^−^ aBC.^9^, ^30^ Ribociclib is the only CDK4/6i to be given a category 1 recommendation by the National Comprehensive Cancer Network (NCCN) guidelines for use in combination with an AI in first‐line treatment of HR^+^/HER2^−^ aBC.^31^ Therefore, ribociclib is to be considered standard of care in first‐line treatment in this indication.^32^

Based on MONALEESA‐2 results, ribociclib was first licensed in the US in March 2017 and in the EU in August 2017 for patients with HR^+^/HER2^−^ aBC in combination with an AI/FUL as initial endocrine‐based therapy, or in women who have received prior endocrine therapy.

After the initial approval, the observational RIBANNA study was initiated to gather real‐world evidence on the effectiveness and safety of ribociclib+AI/FUL utilized in clinical routine practice.

## 2 METHODS

### 2.1 Study design and setting

RIBANNA (registered under NCT06311383) is a prospective, multicenter, observational study to evaluate the safety and effectiveness of ribociclib+AI/FUL in pre‐ and post‐menopausal patients with aBC treated according to the label in 279 specialized breast cancer centers across Germany. However, to cover all therapies in the first‐line setting, also patients treated with CT or ET_mono_ were allowed to be included. The choice of first‐line treatment with ribociclib+AI/FUL, ET_mono_ (e.g., letrozole, anastrozole, fulvestrant) or CT (e.g., taxanes, capecitabine with/without bevacizumab) was to be made independently by the treating physician prior to enrollment.

Enrolled patients were allocated to one of the following three treatment cohorts:

- Ribociclib plus AI/FUL (ribociclib cohort),
- Endocrine monotherapy (ET cohort), or
- Chemotherapy (CT cohort).

All enrolled patients were to be treated according to the Summary of Product Characteristics (SmPC) in their respective valid version. Adult women were eligible for this study, if they had histologically confirmed diagnosis of HR^+^/HER2^−^ aBC without prior systemic treatment for advanced/metastatic disease. Written IC from patients was mandatory before any study‐related activities. First‐line treatment was allowed to be initiated for a maximum of 28 days prior to IC. Minors or incapacitated patients, or patients participating in another clinical study were not included.

All enrolled study patients are followed for up to three treatment lines, until individual study discontinuation or regular study end, whichever occurs first (Supplement‐Figure S1). Data are collected as per site routine treatment or follow‐up schedule until regular study end (February 16,2025), which is defined as 4 years after last patient's first visit (on February 16, 2021).

Since October 2017, annual interim analyses (IAs) have been conducted. This report describes the results from the current 7th IA with cutoff date September 30, 2024.

### 2.2 Study oversight and steering committee

This study was funded by Novartis and conducted in accordance with the ISPE Pharmacoepidemiological Practice Guidelines,^33^ STROBE guidelines,^34^ Joint Recommendations of the Federal Institute for Drugs and Medical Devices (BfArM) and the Paul Ehrlich Institute, and the Declaration of Helsinki.

The study was notified to the BfArM and reported to the concerned Health Insurance Funds.

A steering committee consisting of nine investigators was established for continuous review of study results and guidance on study conduct.

### 2.3 Criteria for evaluation

Criteria for evaluation in RIBANNA cover a broad range of measurements (Supplement‐Table S1). After the third therapy line, if applicable, only OS is tracked. The following core effectiveness and safety endpoints were addressed in the present analysis:

- PFS in first‐line, defined as time from first intake of the respective medication up to first documented progression or death from any cause. Progression was locally determined by investigators; patients without an event were censored at the end of the first therapy line. The rate of patients without disease progression after 18 months in the subset of the ribociclib cohort similar to the MONALEESA‐2 population was used for the sample size calculation in this study.
- PFS2, defined as time from first intake of the respective medication in first‐line up to progression or death after start of second‐line treatment. All patients who started first‐line treatment were included in this analysis; patients without an event in first‐line were censored at the date of the last contact or cut‐off date.
- OS, defined as time from first intake of the respective medication up to death from any cause during the entire study period. If a patient was not known to have died, survival was censored at the date of last contact.
- Frequency and severity of treatment‐emergent adverse events (AEs) on first‐line therapy, defined as events that started or worsened after first intake of ribociclib during first‐line therapy until 30 days after first treatment line.
- Description of reasons for permanent discontinuation of ribociclib treatment in first‐line.

### 2.4 Sample size

Under the assumption of a PFS rate of 54% as was observed at month 18 in the MONALEESA‐2 trial,^15^ the analysis of 850 patients allows a precision of 0.034 in terms of the width of the respective 95% confidence interval (CI). Accounting for the assumption that 50% of enrolled patients would be similar to the MONALEESA‐2 population and the assumed drop‐out rate of 15%–20%, resulted in an effective sample size of 2040 patients in the ribociclib cohort. The planned sample size of 490 patients per ET/CT cohort (408 patients and surplus to address 15%–20% dropouts) was chosen to have at least 20% of the sample size of patients enrolled in the ribociclib cohort to appropriately assess potential baseline differences across the cohorts. The two latter cohorts were to be closed after the enrollment of 490 patients each or following the enrollment of the last patient into the ribociclib cohort, whichever occurred first.

**FIGURE 1:**
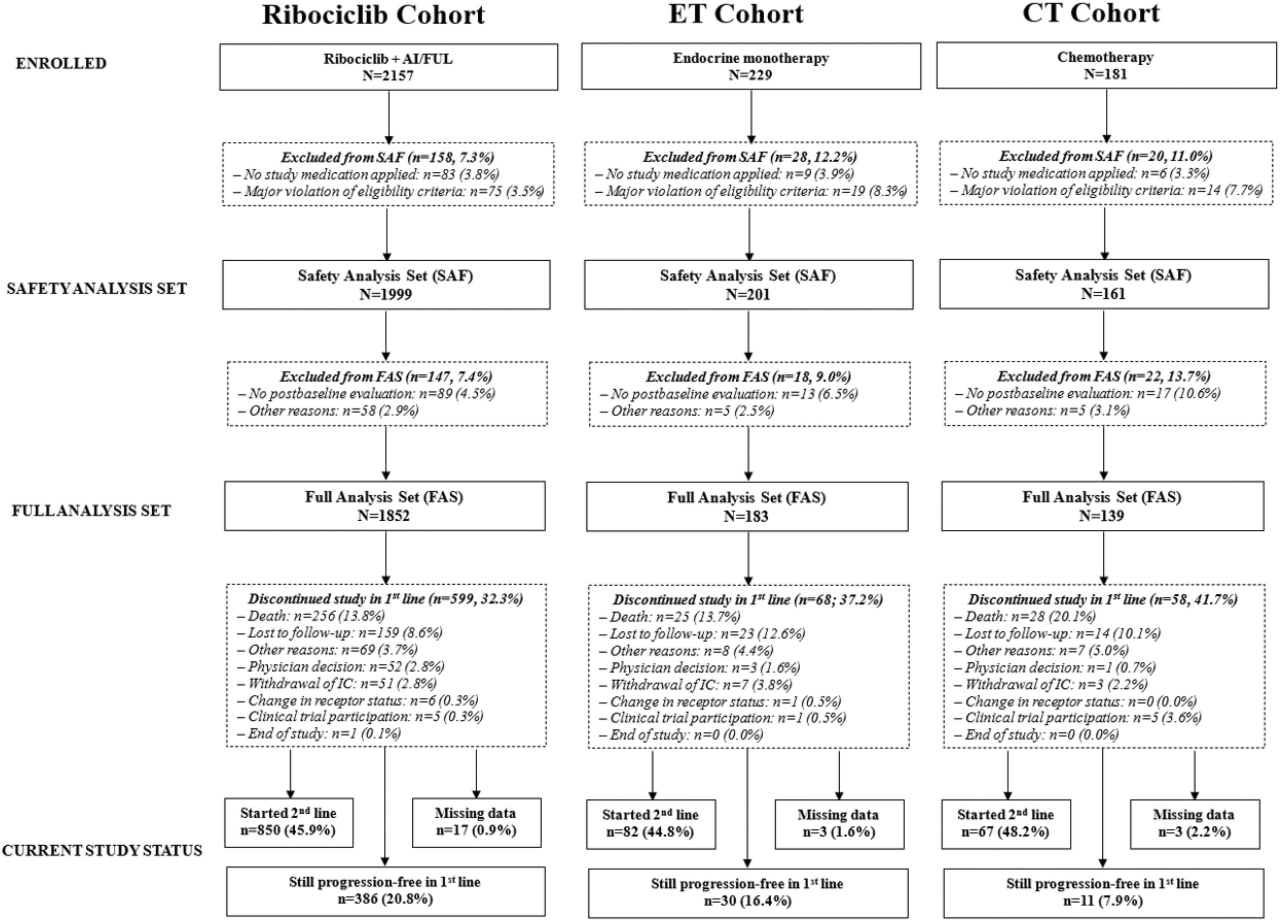
Disposition of study patients enrolled in the RIBANNA study and analysis sets. AI, aromatase inhibitor; CT, chemotherapy; ET, endocrine monotherapy, FAS, full analysis set; FUL, fulvestrant; IC, informed consent; SAF, safety analysis set. Documentation as per investigator's entry on the study end page. The category “other” is an umbrella term for patients with free‐text entries.

Study recruitment was stopped in February 2021 when the ribociclib cohort was fully recruited (*n* = 2157). At that time, recruitment numbers in the ET and CT cohorts were smaller than planned (229 and 181 patients, respectively; Figure 1).

### 2.5 Statistical analysis

#### 2.5.1 General information

The real‐world data sources in this study are routinely maintained medical records on‐site as well as study‐specific paper questionnaires completed by patients.

Due to the observational study design, all analyses and related *p*‐values/95% CIs are considered descriptive.

Analysis was carried out using SAS® v9.4. Categorical variables were summarized by absolute and relative frequencies, while continuous variables were summarized by descriptive statistics. Time‐to‐event data were assessed using the Kaplan–Meier (KM) method.

Therapy lines were primarily classified based on documented progression in case of missing data for changes in therapy line. Each progression served as a surrogate parameter for a switch to the next treatment line. Anti‐tumor medications started after the date of progression were considered next‐line treatment.

The safety analysis set (SAF) comprised all enrolled patients who had received at least one dose of the respective treatment, without any documented protocol deviation leading to exclusion from the analysis if present (as determined by medical review). The full analysis set (FAS) included all patients from the SAF, for whom at least one post‐baseline effectiveness evaluation was recorded.

Safety and effectiveness analyses were performed in the SAF and FAS, respectively. Demographic and other baseline characteristics were summarized for the FAS and, where applicable, tested for baseline differences among treatment cohorts using F‐test or Chi‐square tests.

AEs were coded using the MedDRA v27.0 terminology, and grading of AEs was done by investigators as per site routine according to Common Terminology Criteria for Adverse Events (CTCAE v5.0).

#### 2.5.2 Analysis of time‐dependent effectiveness endpoints

PFS and OS were analyzed using the KM method. Percentiles of the event time distribution (25%, 50%, 75%) are presented along with their two‐sided 95%‐CI.

Additionally, KM estimates for PFS event rates were presented for time points 18 and 24 months (OS: 24 and 48 months). KM curves (with censored patients, number of events, and number of patients at risk) were displayed graphically.

Cox proportional hazard regression was calculated post hoc to adjust for baseline differences that were observed among treatment cohorts (Table 1). Cox model for first‐line PFS considered the covariates “treatment cohort,” “age group,” “previous therapy,” “ECOG performance status,” “grading status,” and “metastasis status” (excluding patients with missing covariate data). These covariates showed significant baseline differences between the cohorts and were considered clinically relevant by steering committee members regarding PFS. Hazard ratios (HRs) with ribociclib as reference were calculated, and adjusted survival curves for PFS were produced from proportional hazard models with the adjusted group prognosis approach. In addition, the associated PFS KM estimates were derived. The adjusted survival curves were displayed together with unadjusted KM curves by cohort.

**TABLE 1:** Selected demographic and other baseline characteristics in the RIBANNA treatment cohorts (FAS).

		Ribociclib + AI/FUL (*N* = 1852)	Endocrine monotherapy (*N* = 183)	Chemotherapy (*N* = 139)	*p*‐value^a^
Age (years)	Mean ± SD	65.5 ± 11.6	71.2 ± 11.2	61.1 ± 10.9	*<.001*
	Median	66.0	74.0	61.0	
	Range	27.0–92.0	39.0–92.0	34.0–85.0	
Age groups, *n* (%)^b^	<75 years	1356 (73.2)	95 (51.9)	124 (89.2)	*<.001*
	75–≤80 years	341 (18.4)	46 (25.1)	14 (10.1)	
	>80 years	155 (8.4)	42 (23.0)	1 (0.7)	
Menopausal status, *n* (%)	Pre‐menopausal	142 (7.7)	5 (2.7)	12 (8.6)	.*066*
	Peri‐menopausal	51 (2.8)	3 (1.6)	6 (4.3)	
	Post‐menopausal	1642 (88.7)	173 (94.5)	119 (85.6)	
	Missing data	17 (0.9)	2 (1.1)	2 (1.4)	
Baseline ECOG‐PS, *n* (%)^b^	0	823 (44.4)	62 (33.9)	59 (42.4)	*<.001*
	1	643 (34.7)	72 (39.3)	51 (36.7)	
	>1	134 (7.2)	29 (15.8)	16 (11.5)	
	Missing data	252 (13.6)	20 (10.9)	13 (9.4)	
Grading, *n* (%)^b^	G1 + G2	1209 (65.3)	118 (64.5)	73 (52.5)	.*001*
	G3	476 (25.7)	35 (19.1)	53 (38.1)	
	Missing data	167 (9.0)	30 (16.4)	13 (9.4)	
Locally advanced disease, *n* (%)	No	480 (25.9)	60 (32.8)	23 (16.5)	*Not tested*
	Yes	1364 (73.7)	123 (67.2)	116 (83.5)	
	Missing data	8 (0.4)	0 (0.0)	0 (0.0)	
Any metastases at study start, *n* (%)	No	47 (2.5)	1 (0.5)	6 (4.3)	*Not tested*
	Yes	1792 (96.8)	181 (98.9)	133 (95.7)	
	Missing data	13 (0.7)	1 (0.5)	0 (0.0)	
Grouped metastases at study start, *n* (%)^b^	CNS, liver, lung	762 (41.1)	47 (25.7)	92 (66.2)	*<.001*
	Bone only	552 (29.8)	89 (48.6)	17 (12.2)	
	Skin, lymph nodes, other	447 (24.1)	42 (23.0)	21 (15.1)	
	Missing data	91 (4.9)	5 (2.7)	9 (6.5)	
Treatment‐free interval, *n* (%)	Missing information for performed prior antineoplastic therapy	21 (1.1)	0 (0.0)	1 (0.7)	*0.456*
	TFI >12 months	554 (29.9)	60 (32.8)	41 (29.5)	
	TFI ≤12 months	416 (22.5)	32 (17.5)	24 (17.3)	
	De novo^c^	658 (35.5)	63 (34.4)	51 (36.7)	
	Missing data	203 (11.0)	28 (15.3)	22 (15.8)	
Any prior chemotherapy, *n* (%)	No	1160 (62.6)	125 (68.3)	93 (66.9)	*0.212*
	Yes	692 (37.4)	58 (31.7)	46 (33.1)	
Previous antineoplastic therapy^b^	Endocrine therapy	269 (14.5)	31 (16.9)	17 (12.2)	*0.323*
	Chemotherapy	306 (16.5)	21 (11.5)	24 (17.3)	
	Endocrine therapy + Chemotherapy	386 (20.8)	37 (20.2)	22 (15.8)	
	No previous therapy^d^	891 (48.1)	94 (51.4)	76 (54.7)	

Abbreviations: AI, Aromatase inhibitor; CNS, central nervous system; FAS, full analysis set; FUL, fulvestrant; PS, performance status; SD, standard deviation; TFI, treatment‐free interval.

^a^ Global *p*‐values derived from F‐test (continuous data) or Chi‐square test (categorical data) for inhomogeneity (differences) across the 3 cohorts.

^b^ These variables were used for adjustment in the Cox proportional hazard regression model.

^c^ Patients who did not receive any prior antineoplastic treatment.

^d^ Due to de novo disease or missing information about adjuvant treatment.

In patients receiving first‐line ribociclib, post hoc PFS subgroup analyses were performed for age, ECOG‐PS baseline score, grading at initial diagnosis, site of internal organ metastases at the start of the study, treatment‐free interval (TFI) and relative dose intensity. Since this was an exploratory analysis, no formal statistical analyses comparing the subgroups were performed. The relative dose intensity was calculated for each patient as (actual average dose intensity/planned dose intensity) × 100 in percent. The actual average dose intensity was calculated for each patient as the total sum of all daily doses divided by the number of days with planned ribociclib intake in first‐line. To do this, the duration of treatment in days was divided by 28 (= number of days per cycle) and multiplied by 21 (= days with intake per cycle). The planned dose intensity corresponded to 600 mg for 3 weeks followed by 1 week off treatment. For the subgroup analysis, the 30th percentile, 60th percentile, and 90th percentile were compared. This corresponds to a relative dose intensity of 1.96%–69.76% (30th percentile), 70%–99.91% (60th percentile), and 100%–104.76% (90th percentile).

## 3 RESULTS

### 3.1 Patient disposition and current duration of observation

This analysis included data from enrollment of the first patient on October 9, 2017 until the data cutoff on September 30, 2024. The median duration of follow‐up was 2.8 years and ranged from 0.0 to 6.9 years.

Out of 2567 enrolled patients in total (2157 in the ribociclib cohort), 1999 patients were included in the ribociclib cohort SAF (enrolled 229 in the ET and 181 in the CT cohort; SAF 201 in the ET and 161 in the CT cohort, respectively). The FAS consisted of 1852 patients in the ribociclib cohort, 183 in the ET cohort, and 139 in the CT cohort (Figure 1). Among patients included in the FAS, rates for study discontinuation in first‐line due to any reason were lower in the ribociclib cohort than in the ET and CT cohorts (32.3% vs. 37.2% and 41.7%, respectively); in each cohort, the most common reason for study discontinuation in first‐line was “death” (ribociclib: 13.8%, ET: 13.7%, CT: 20.1%). Approximately half of all patients from the FAS had started second‐line treatment at the data cutoff (ribociclib: 45.9%, ET: 44.8%, 48.2%) and at that time, the ribociclib cohort had the highest proportion of patients who were still progression‐free in first‐line (20.8% vs. 16.4% and 7.9%, respectively).

### 3.2 Patient baseline characteristics

In the ribociclib cohort, patients' mean age was 65.5 ± 11.6 years (range: 27.9–92.9), 7.7% were pre‐menopausal and 2.8% peri‐menopausal, 7.2% had ECOG‐PS >1, 25.7% had grade 3 (G3) histology, 96.8% showed metastases, and 73.7% presented with locally advanced disease (Table 1).

Preceding adjuvant therapy was reported in 961 patients (51.9%) in the ribociclib cohort, 98 patients (53.6%) in the ET cohort, and 66 patients (47.5%) in the CT cohort. Documented prior antineoplastic treatments in the ribociclib cohort included ET (14.5%), CT (16.5%), and ET + CT (20.8%), while 48.1% had not received prior therapy, with 35.5% being treated de novo (Table 1).

#### 3.2.1 Utilization of ribociclib

Combination partners for ribociclib in first‐line (SAF, *N* = 1999) were letrozole (67.3%), fulvestrant (21.1%), anastrozole (5.6%), and exemestane (4.5%).

Median duration of first‐line treatment with ribociclib was 453 days (range: 1.0–2516). Starting doses were 600 mg/day in 1777 patients (88.9%), 400 mg/day in 132 patients (6.6%), and 200 mg in 79 patients (4.0%). Mean daily doses (excluding interruption periods) were 513.8 ± 114.3 mg/day (median: 600.0 mg/day).

Of the patients starting second‐line treatment, 132 patients (15.5%) in the ribociclib cohort, 20 patients (24.4%) in the ET cohort, and 7 patients (10.4%) in the CT cohort received ribociclib in combination with AI/FUL (Supplement‐Table S2).

### 3.3 Effectiveness outcomes

#### 3.3.1 PFS in first‐line and OS observed in the ribociclib cohort

**FIGURE 2:**
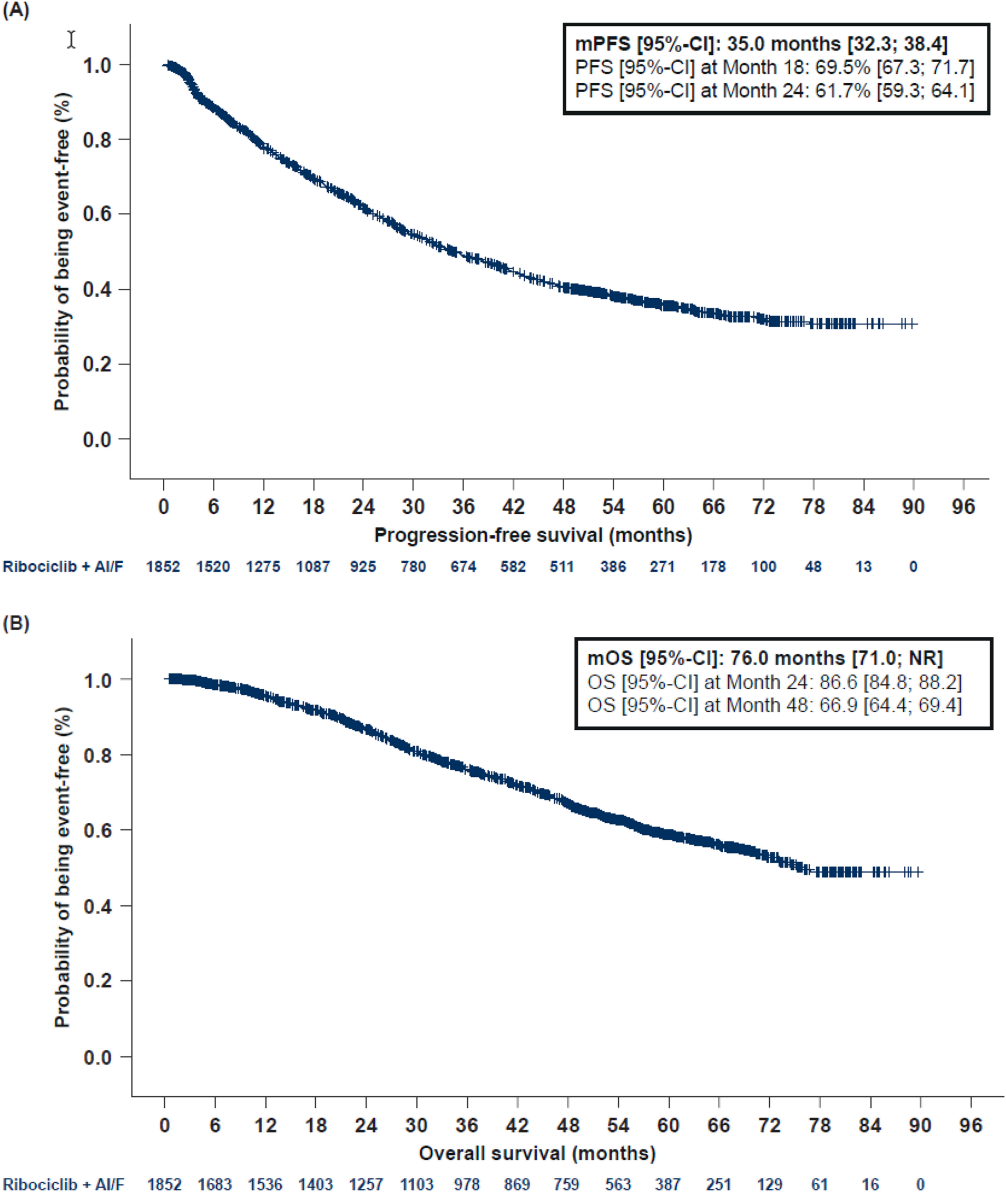
Progression‐free survival (in first‐line) and overall survival in the ribociclib cohort (FAS). AI, aromatase inhibitor; CI, confidence interval; F, fulvestrant; FAS, full analysis set; mPFS, median progression‐free survival; mOS, median overall survival; NR, not reached.

Both mPFS and mOS were reported in the RIBANNA study in all treatment cohorts during the observation time of the study. In the ribociclib cohort, mPFS was 35.0 months (95%‐CI: [32.3; 38.4]). Median OS was 76.0 months (95%‐CI: [71.0; not reached]; Table 2 and Figure 2).

**TABLE 2:** Progression‐free survival and overall survival on first‐line treatment in the total ribociclib cohort (FAS).

	Progression‐free survival	Overall survival
No. of censored observations	853	1278
No. of events	999	574
KM estimator for time to event (months)		
75%‐Quantile [95%‐CI]	14.2 [12.8; 15.6]	37.6 [34.4; 41.0]
50%‐Quantile [95%‐CI]	35.0 [32.3; 38.4]	76.0 [71.0; NR]
25%‐Quantile [95%‐CI]	NR	NR
KM estimator for time to event (%) at		
Month 18 (OS: 24) after reference time point [95%‐CI]	69.5 [67.3; 71.7]	86.6 [84.8; 88.2]
Month 24 (OS: 48) after reference time point [95%‐CI]	61.7 [59.3; 64.1]	66.9 [64.4; 69.4]

*Note*: Median follow‐up was 2.9 years in the ribociclib + AI/FUL cohort.

Abbreviation: CI, confidence interval; FAS, full analysis set; KM, Kaplan Meier; NR, not reached; OS, overall survival.

#### 3.3.2 PFS outcomes observed in the parallel treatment cohorts

**FIGURE 3:**
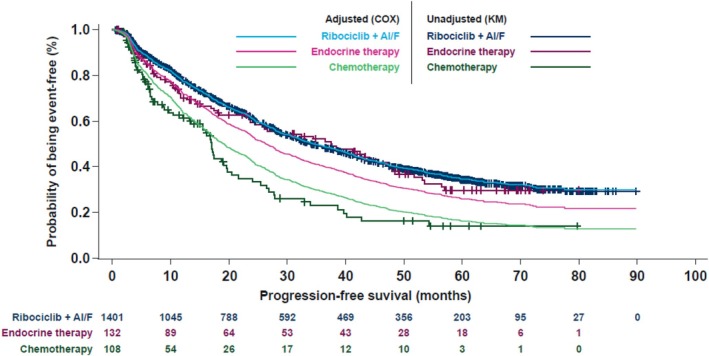
Progression‐free survival observed in the three treatment cohorts (Kaplan–Meier plots and Cox proportional hazard regression adjusted for baseline covariates) (FAS). AI, aromatase inhibitor; COX, cox hazard regression model; CT, chemotherapy; ET, endocrine monotherapy; F, fulvestrant; FAS, full analysis set; KM, Kaplan–Meier.

The mPFS in the ET and CT cohorts based on KM estimates were 37.4 months and 17.0 months, respectively (Supplement‐Table S3). Baseline differences across the three cohorts were seen for, for example, age, ECOG‐PS, grading, and pattern of metastases (global *p*‐value for differences across cohorts <.001; Table 1). To assess the impact of these differences, a post hoc Cox regression model was adjusted for relevant baseline factors. The resulting adjusted PFS curves from the model are displayed together with the unadjusted KM plots for PFS in the three cohorts (Figure 3). After adjusting for baseline characteristics, the ribociclib cohort revealed longer PFS vs. the ET cohort, suggesting a benefit for addition of ribociclib. Consistently, the adjustment resulted in a descriptive mPFS from 35.0 months (unadjusted, KM) to 34.7 months (adjusted) in the ribociclib cohort, from 37.4 to 26.4 months in the ET cohort, and from 17.0 months to 19.2 months in the CT cohort. Hazard ratio for the comparison of ET with ribociclib (as reference) was HR = 1.288 (95%‐CI: [1.006; 1.649]; *p* = .0451) and for CT versus ribociclib it was HR = 1.776 (95%‐CI: [1.377; 2.290]; *p* <.0001), both in favor of ribociclib.

#### 3.3.3 Subgroup analysis of factors affecting PFS in the ribociclib cohort

To provide additional insights on whether some patients benefit more from ribociclib therapy than others, exploratory subgroup analyses for age, ECOG‐PS, grading at initial diagnosis, site of internal organ metastases, and TFI as well as relative dose intensity were performed. Differences were observed between the two age groups, >65 and ≤65 years, where the benefit of first‐line ribociclib seemed to be greater in older patients: mPFS was 28.0 months (95%‐CI: [24.9; 30.8]) for patients ≤65 years versus 43.6 months (95%‐CI: [39.7; 49.8]) for patients >65 years (Supplement‐Figure S2, panel A). PFS was comparable between the three analyzed ECOG‐PS subgroups: mPFS was 38.8 months (95%‐CI: [32.7; 43.5]) for patients with an ECOG‐PS score of 0 at baseline versus 33.0 months (95%‐CI: [29.0; 39.3]) for an ECOG‐PS score of 1 versus 35.8 months (95%‐CI: [27.6; 54.9]) for an ECOG‐PS score of >1 (Supplement‐Figure S2, panel B). Patients with grade 3 (G3) histology at diagnosis had initially worse PFS outcomes than those with grade 1 and 2 (G1 + G2): mPFS was 38.0 months (95%‐CI: [33.7; 41.5]) for G1 + G2 versus 26.3 months (95%‐CI: [22.5; 30.5]) for G3. However, the difference in PFS evened out over time (Supplement‐Figure S2, panel C). Patients with central nervous system (CNS), liver, or lung metastases initially had worse PFS outcomes compared to patients with metastases at other sites: mPFS was 27.1 months (95%‐CI: [23.7; 31.1]) for CNS, liver, or lung metastases vs. 38.8 months (95%‐CI: [33.6; 46.0]) for bone only metastases and 40.5 months (95%‐CI: [33.7; 47.4]) for skin, lymph node, and other metastases (Supplement‐Figure S2, panel D). Patients with short TFI, expectedly had worse PFS outcomes than those with long TFI: mPFS was 17.9 months (95%‐CI: [15.0; 20.4]) for TFI≤12 months versus 40.1 months (95%‐CI: [32.6; 45.8]) for TFI >12 months (Supplement‐Figure S2, panel E). PFS was not negatively affected in patients with reduced ribociclib relative dose intensity: mPFS was 40.9 months (95%‐CI: [35.6; 45.2]) in patients with an average dose intensity of 1.96%–69.76% (30th percentile) versus 40.5 months (95%‐CI: [35.5; 46.9]) in patients with an average dose intensity of 70%–99.91% (60th percentile) versus 28.6 months (95%‐CI: [25.4; 32.7]) in patients with an average dose intensity of 100%–104.76% (90th percentile) (Supplement‐Figure S2, panel F).

#### 3.3.4 Exploratory analysis of PFS separated by post hoc‐created subgroups within the ribociclib cohort

To allow a more targeted descriptive comparison of PFS with the pivotal MONALEESA‐2, −3 and −7 trials, the ribociclib patients in RIBANNA were selected and, if possible, assigned to one of three post hoc created MONALEESA (ML)‐like subgroups. The allocation was based on a reconciliation of selected key eligibility criteria of the respective pivotal trials (criteria for allocation are summarized in Supplement‐Table S5). 560 of the 1852 ribociclib patients were assigned to the ML2‐like subgroup, 203 to the ML3‐like subgroup, and 59 to the ML7‐like subgroup. 1030 patients could not be attributed to a certain ML‐like subgroup due to missing data or patient characteristics that did not match the selected eligibility criteria of one of the three pivotal trials. These patients formed the fourth subgroup of non‐attributable patients. Within these four subgroups, mPFS was 43.9 months (95%‐CI [36.4; 49.5]) in the ML2‐like subgroup, 22.9 months (95%‐CI [18.0; 25.8]) in the ML3‐like subgroup, 30.3 months (95%‐CI [16.1; not reached]) in the ML7‐like subgroup, and 35.5 months (95%‐CI [31.7; 38.6]) in the group of non‐attributable patients (Supplement‐Table S6).

#### 3.3.5 Supplementary analysis of PFS2 in the ribociclib cohort

PFS2 (i.e., progression‐free survival from first intake of the respective medication until progression or death in second line) was calculated in addition to PFS to deepen insight into the longer‐term course of disease. A total of 850 patients (45.9%) in the ribociclib cohort had switched to second‐line treatment at cutoff date (Figure 1). The most common subsequent antineoplastic medications were chemotherapy (303 patients, 35.6%) and endocrine monotherapy (227 patients, 26.7%). In total, the calculation of PFS2 in the ribociclib cohort (FAS) showed an mPFS2 of 52.5 months (95%‐CI: [49.2; 56.9]); PFS2 at Month 24 was 76.7% (95%‐CI: [74.5; 78.7]) (Supplement‐Table S7 and Supplement‐Figure S3).

#### 3.3.6 AEs occurring in the ribociclib cohort

The pattern of the most common AEs (Table 3) occurring on first‐line treatment in the ribociclib cohort included cytopenia (e.g., neutropenia [27.1% of SAF patients], leukopenia [21.7%], anemia [10.0%]), gastrointestinal complaints (e.g., nausea [26.4%], diarrhea [13.4%], constipation [9.5%], vomiting [9.0%]), general conditions (e.g., fatigue [26.0%], dizziness [9.3%], headache [7.5%]), skin manifestations (alopecia [15.8%], general physical health deterioration (8.9%), rash [8.3%], hot flush [7.7%], pruritus [7.4%]) and mainly reflected the established side effect profile of ribociclib in combination with AI/FUL, while malignant neoplasm progression (9.7%) was likely attributable to the underlying disease.

**TABLE 3:** Overview of adverse events and most common adverse events occurring on first‐line treatment in the ribociclib cohort (SAF).

	Ribociclib + AI/FUL
	(*N* = 1999)
	*n* (%)
Patients with any AEs	1849 (92.5)
Patients with any SAEs	865 (43.3)
Patients with all related SAEs^a^	417 (20.9)
Patients with ribociclib‐related SAEs	267 (13.4)
Deaths	317 (15.9)
Most common AEs at the PT level^b^	
Neutropenia	541 (27.1)
Nausea	527 (26.4)
Fatigue	520 (26.0)
Leukopenia	434 (21.7)
Alopecia	316 (15.8)
Diarrhea	268 (13.4)
Arthralgia	241 (12.1)
Dyspnoea	210 (10.5)
Anemia	200 (10.0)
Malignant neoplasm progression	194 (9.7)
Constipation	190 (9.5)
Dizziness	185 (9.3)
Vomiting	179 (9.0)
General physical health deterioration	177 (8.9)
Bone pain	167 (8.4)
Rash	165 (8.3)
Back pain	155 (7.8)
Hot flush	154 (7.7)
Headache	149 (7.5)
Pruritus	147 (7.4)

Abbreviations: AE, adverse event; AI, aromatase inhibitor; FUL, Fulvestrant; PT, Preferred term; SAE, serious adverse event; SAF, safety analysis set.

^a^ Regarded as related (investigator's assessment) to any of the study drugs (i.e., ribociclib, AI, fulvestrant).

^b^ Listed are the 20 most common adverse events reported in the ribociclib cohort during first‐line treatment.

SAEs considered related to ribociclib (investigator's assessment) occurred in 267 patients (13.4%) and included (incidence cut: 0.5%; equal to 9 patients) general physical health deterioration (33 patients, 1.7%), neutropenia (23 patients, 1.2%), anemia (16 patients, 0.8%), dyspnea (16 patients, 0.8%), pneumonia (15 patients, 0.8%), leukopenia (14 patients, 0.7%), malignant neoplasm progression (14 patients, 0.7%), diarrhea (13 patients, 0.7%), nausea (11 patients, 0.6%), hepatotoxicity (11 patients, 0.6%), liver function test increased (11 patients, 0.6%), vomiting (9 patients, 0.5%), and death (9 patients, 0.5%).

Adverse events of special interest (AESIs) included ALT increased (83 patients, 4.2%), AST increased (80 patients, 4.0%), blood bilirubin increased (13 patients, 0.7%), hyperbilirubinemia (2 patients, 0.1%), COVID‐19 (126 patients, 6.3%), nasopharyngitis (127 patients, 6.4%), urinary tract infection (108 patients, 5.4%), anemia (200 patients, 10.0%), leukopenia (434 patients, 21.7%), neutropenia (541 patients, 27.1%), thrombocytopenia (120 patients, 6.0%), acute kidney injury (25 patients, 1.3%), renal failure (38 patients, 1.9%), urinary retention (21 patients, 1.1%), and QT interval prolongation (78 patients, 3.9%; Supplement‐Table S8).

Treatment discontinuation of the ribociclib regimen cohort due to AEs in first‐line was reported in 24 patients (1.2%; Supplement‐Table S9), while dose reduction of ribociclib due to AEs occurred in 234 patients (11.7%).

## 4 DISCUSSION

### 4.1 PFS and OS on treatment with ribociclib in a real‐world setting

A central finding in the RIBANNA study was that first‐line treatment with ribociclib+AI/FUL in clinical routine practice resulted in an mPFS of 35.0 months and an mOS of 76.0 months. These results exceeded the first‐line mPFS outcomes that were reported from the pivotal trials MONALEESA‐2 (25.3 and 63.9 months), MONALEESA‐3 (33.6 and 67.6 months) and MONALEESA‐7 (23.8 and 58.7 months; Supplement‐Table S4). Thus, the beneficial results seen in the pivotal clinical trials were supported by the RIBANNA study also in the real‐world setting.

The post hoc analyses by creating ML‐like subgroups as subsets of the total ribociclib cohort indicated that the mPFS in the total ribociclib cohort was mainly driven by the performance of the ML2‐like patients (mPFS = 43.9 months [*N* = 560] vs. 25.3 months in MONALEESA‐2) and the ML7‐like patients (mPFS = 30.3 months [*N* = 59] vs. 23.8 months in MONALEESA‐7). In contrast, the mPFS among the ML3‐like patients was shorter than in the first‐line analysis of MONALEESA‐3 (22.9 months [*N* = 203] vs. 33.6 months) or in the two other ML‐like subgroups. This effect might be attributed to routine clinical practice of prescribing ribociclib plus fulvestrant for patients with poorer prognosis, including patients with early relapse. This assumption is supported by the large proportion of patients with TFI ≤12 months in the ML3‐like subgroup (41.9%) compared with the ML2 and ML‐7 like subgroups (12.3% and 30.5%, respectively; Supplement‐Table S10).

Importantly, the large group of patients that could not be attributed to one of the ML‐like cohorts (*N* = 1030; either due to missing information or because they would not have been eligible for the pivotal trials) showed an mPFS of 35.5 months, suggesting that ribociclib provided benefit even to a patient population beyond those included in the pivotal trials and still eligible for ribociclib treatment as per SmPC.

The analysis of PFS2 (i.e., considering the time including second‐line treatment, if applicable) reflected the trend toward better PFS outcomes in the ribociclib cohort, since the observed mPFS2 was 52.5 months and, thus, higher than those reported from the pivotal trials MONALEESA‐2 (39.8 months^35^), MONALEESA‐3 (50.7 months^22^), and MONALEESA‐7 (44.2 months^25^).

Generally, descriptive comparisons between RIBANNA and MONALEESA outcomes should be interpreted with caution, since RIBANNA was not designed to allow an indirect cross study comparison with the pivotal trials. Partly different patient populations were investigated in RIBANNA and MONALEESA due to the broad label of ribociclib. RIBANNA allowed the enrollment of patients with ECOG >1, CNS metastases, and endocrine resistance, that is, there were no restrictions for disease‐free interval (DFI) <12 months. Comparison of baseline characteristics showed that more patients with de novo disease and DFI <12 months (TFI in RIBANNA) and less patients with visceral disease were included in RIBANNA than in the MONALEESA trials. Moreover, RIBANNA patients were on average older than patients in the MONALEESA trials. Overall, these differences in enrollment criteria and in baseline characteristics might explain the numerical differences seen in the PFS and OS outcomes between RIBANNA and the MONALEESA trials. Other potential reasons for the performance seen in the ML‐like cohorts compared with the respective trials might include: Currently unidentified confounding factors, a more effective general disease management developed over recent years, higher familiarity and more experience with the ribociclib regimen gained over time, the accuracy of PFS assessment was determined, and differences in sample sizes. Overall, the RIBANNA study reflected the PFS and OS benefits in first‐line therapy that were previously demonstrated in the pivotal trials.

Exploratory subgroup analyses regarding age, ECOG‐PS, grading at initial diagnosis, metastasis site, and TFI showed that PFS outcomes in these subgroups were consistent with the respective risk prognoses and suggested beneficial effects in all subgroups. As expected, worse mPFS was observed for patients with known adverse prognostic factors: age ≤65 years; G3 histology; CNS, liver, or lung metastases; and short TFI. Interestingly, for G3 histology vs. G1 + G2, the difference in PFS evened out over time—this finding is in line with a recently published analysis of RIBANNA that investigated conditional PFS (reflecting patient prognosis after initial management) both in the whole ribociclib cohort as well as in selected subgroups, among them grading at initial diagnosis.^36^ In contrast, no relevant differences in mPFS were observed between the ECOG‐PS subgroups.

Lowering the relative ribociclib dose intensity did not lead to worse PFS outcomes in our exploratory subgroup analysis. This finding corroborates the results of a previously published analysis regarding the safety and impact of dose reductions on efficacy in pooled data from the randomized MONALEESA‐2, ‐3 and ‐7 clinical trials. The pooled analysis showed that the clinical benefit of ribociclib treatment was preserved when dose modifications were undertaken: median PFS was 24.8, 24.9, and 29.6 months for patients who received ≤71% (30th percentile), 72%–96% (60th percentile), and 97%–100% (90th percentile) ribociclib relative dose intensity, respectively.^37^ Seeing similarly no deleterious effect of dose reductions on efficacy in the real‐world data from RIBANNA is a reassuring finding for physicians and patients who need to undergo dose reductions, for example, due to AEs. And although it seems that in RIBANNA, PFS outcomes were, in fact, more favorable in patients who received lower relative ribociclib dose intensities, caution should be exercised when interpreting these results: RIBANNA is not a randomized trial and therefore does not allow conclusions regarding causality for the observed association between dose intensity and outcomes: the observed longer mPFS with lower ribociclib dose intensities could be due to differences in patient characteristics between the dose intensity subgroups; in fact, we found that patients in the lowest dose intensity percentile were on average older than those in the other percentiles (mean age 68.4 years, 64.8 years, and 63.4 years for the 30th, 60th, and 90th percentile, respectively) and the proportion of older patients >65 years was significantly higher (64.2%, 51.1%, and 42.8%, respectively). Furthermore, in the randomized AMALEE trial that included pre‐ and postmenopausal patients with HR+/HER2− aBC with no prior therapy for aBC, reducing the starting dose of ribociclib from the recommended dose of 600 mg to 400 mg/day led to a reduction of the overall response rate. As a result, the trial failed its primary endpoint and was unable to demonstrate noninferiority of the lower ribociclib dose.^38^

Meanwhile, the label for ribociclib combined with an AI was extended to adjuvant treatment of HR^+^/HER2^−^ patients with early breast cancer at high risk of recurrence on the basis of the recently published NATALEE trial.^39^

### 4.2 PFS outcomes on first‐line treatment with ET and CT in the parallel cohorts

While the patient enrollment goal was achieved for the ribociclib cohort (2.157 patients), the recruitment into the two additional ET and CT treatment cohorts (229 and 181 patients, respectively) was lower than intended, which was probably caused by the evolution of CDK4/6i + ET therapy as the standard treatment of choice in first‐line treatment of patients with HR^+^/HER2^−^ aBC.

The observed baseline differences across the three (non‐randomized) treatment cohorts reflected the treatment decisions in routine care that were made prior to study entry. ET was preferably administered to elderly patients with bone metastases only, while CT was preferably chosen for younger patients with G3 histology and visceral disease.

A post hoc comparison of PFS across cohorts was done for exploratory purposes. To account for the observed baseline differences, a Cox regression model adjusted for relevant baseline factors was calculated in addition to the regular KM analyses to allow for a more suitable comparison of PFS outcomes across cohorts. The adjusted mPFS data derived from the Cox regression model indicated descriptive differences in favor of the ribociclib cohort (34.7 months) compared to ET (26.4 months) or CT (19.2 months). Consistently, the HRs from the adjusted model for the comparison of ET versus ribociclib (HR = 1.288; 95%‐CI: [1.006; 1.649]) or CT versus ribociclib (HR = 1.776; 95%‐CI: [1.377; 2.290]) were in favor of ribociclib, and the related 95%‐CIs indicated nominally significant differences. To strengthen the validity of the Cox regression model analysis, a propensity‐score matched analysis would have been desirable. However, propensity‐score matching was not feasible in this case. The number of patients in the ET and CT cohorts was much smaller than originally intended, likely caused by CDK4/6i + ET therapy becoming the standard of care soon after RIBANNA commenced. Furthermore, significant differences in baseline characteristics are apparent between the three cohorts. This would have led to a large loss of data during the matching process, thereby compromising the statistical validity of the analysis.

These data suggest that the combination of ribociclib with AI/FUL in first‐line provides increased PFS benefit when compared to ET or CT alone. However, it must be taken into account that this is not a randomized comparison. Reassuringly, the observations for ribociclib+AI/FUL versus ET are consistent with those from the three randomized MONALEESA trials: across these, mPFS was significantly longer with ribociclib+ET versus ET alone (MONALEESA‐2: 25.3 vs. 16.0 months [HR = 0.57]^21^; MONALEESA‐3: 20.5 versus 12.8 months [HR = 0.59]^17^; MONALEESA‐7: 23.8 versus 13.0 months [HR = 0.55]^19^). In addition, significant benefits were seen for the secondary endpoint OS in all three trials (MONALEESA‐2: 63.9 vs. 51.4 months [HR = 0.76, 95%‐CI: 0.63–0.93]^20^; MONALEESA‐3: 53.7 vs. 41.5 months [HR = 0.73, 95%‐CI 0.59–0.90]^24^; MONALEESA‐7: 58.7 vs. 48.0 months [HR = 0.76, 95%‐CI 0.61–0.96]^25^).

### 4.3 Safety outcomes in first‐line with ribociclib

Consistent with the AE pattern observed in pivotal trials, the most common AEs in RIBANNA were neutropenia (27.1%), nausea (26.4%), fatigue (26.0%), and leukopenia (21.7%). These incidences were clearly smaller than in the pivotal studies e.g., in MONALEESA‐2 after a median follow‐up of only 15.3 months (neutropenia: 74.3%, nausea: 51.5%, fatigue: 36.5%, leukopenia: 32.9%^15^).

AEs of special interest with Grade 3/4 were infrequent and, apart from neutropenia (339 patients, 17.0%), leukopenia (128 patients, 6.4%), and anemia (56 patients, 2.8%), occurred in less than 2.5% of patients. Only a few patients discontinued first‐line treatment in the ribociclib cohort due to AEs or required ribociclib dose reductions due to AEs (1.2% and 11.7%, respectively), while these proportions were higher in MONALEESA‐2 (7.5% and 50.6%, respectively^15^). These differences can most likely be explained by underreporting.

No unexpected or otherwise critical safety signals occurred during the study course, suggesting that ribociclib treatment in a real‐world setting is safe and well‐tolerated.

### 4.4 Overall conclusions

The results of the observational RIBANNA study showed PFS and OS outcomes on first‐line treatment with ribociclib+AI/FUL that supported the beneficial effects of ribociclib demonstrated in the pivotal clinical trials also in a real‐world setting. The safety profile of ribociclib was fully consistent with the established reference safety information. The RIBANNA study confirms that ribociclib unfolds its beneficial effects in a real‐world setting even in patients who were not covered by the pivotal trials.

## AUTHOR CONTRIBUTIONS

**Peter A. Fasching:** Conceptualization; investigation; writing – original draft; writing – review and editing; supervision. **Cosima Brucker:** Investigation; writing – review and editing. **Thomas Decker:** Conceptualization; investigation; writing – review and editing. **Anne Engel:** Data curation; formal analysis; methodology; software; writing – review and editing. **Thomas Göhler:** Investigation; writing – review and editing. **Christian Jackisch:** Investigation; writing – review and editing. **Jan Janssen:** Investigation; writing – review and editing. **Andreas Köhler:** Investigation; writing – review and editing. **Kerstin Lüdtke‐Heckenkamp:** Investigation; writing – review and editing. **Diana Lüftner:** Investigation; writing – review and editing. **Frederik Marmé:** Investigation; writing – review and editing. **Marion van Mackelenbergh:** Investigation; writing – review and editing. **Beate Rautenberg:** Investigation; writing – review and editing. **Marcus Schmidt:** Conceptualization; investigation; writing – review and editing. **Rudolf Weide:** Investigation; writing – review and editing. **Pauline Wimberger:** Investigation; writing – review and editing. **Elena Kisseleff:** Conceptualization; data curation; project administration; supervision; visualization; writing – original draft; writing – review and editing. **Christina Pfister:** Conceptualization; writing – original draft; writing – review and editing. **Claudia Quiering:** Methodology; writing – review and editing. **Christian Roos:** Conceptualization; writing – review and editing. **Achim Wöckel:** Conceptualization; investigation; supervision; writing – review and editing.

## FUNDING INFORMATION

The RIBANNA study and this work is funded by Novartis Pharma GmbH, Nuremberg, Germany (no grant number).

## CONFLICT OF INTEREST STATEMENT

Peter A. Fasching reports personal fees from Novartis, grants from Biontech, grants and personal fees from Pfizer, personal fees from Daiichi‐Sankyo, personal fees from Astra Zeneca, personal fees from Eisai, personal fees from Merck Sharp & Dohme, grants from Cepheid, personal fees from Lilly, personal fees from Seagen, personal fees from Roche, personal fees from Agendia, personal fees from Gilead, personal fees from Mylan, personal fees from Menarini, personal fees from Veracyte, personal fees from Guardant Health, during the conduct of the study; and Translational Research in Oncology (TRIO). Thomas Decker received advisory board honoraria from Novartis, Roche, Lilly, Astra Zeneca, Seagen, Daiichi Sankyo, Pfizer, Iomedico. Anne Engel is an employee of the CRO commissioned to carry out the RIBANNA study. Christian Jackisch has been a consultant for Astra Zeneca, Novartis, Roche; received honoraria from Agendia, Astra Zeneca, ExactSciences, Gilead, GynUpdate, Lilly, Novartis, Pfizer, Roche, StreamedUp; received travel funding from Agendia, Astra Zeneca, Lilly, Pfizer, Gilead, ExactSciences, GynUpdate, Novartis, Roche, StreamedUp. Jan Janssen received personal fees from Astra Zeneca, Beigene, BMS, Ipsen, Johnson and Johnson, Novartis, Octapharm, Stemline, Pfizer, Roche. Andreas Köhler received advisory board honoraria from Novartis (local PI) and Roche (local PI), has been a consultant for Amgen, Astra Zeneca, Daiichi Sankyo; owns shares from Roche; received research funding from Astra Zeneca, GBG, Iomedico, Novartis, Roche, WSG. Kerstin Lüdtke‐Heckenkamp received personal fees from Astra Zeneca, Daiichi Sankyo, Gilead, Lilly, Menarini Stemline, MSD, Novartis, Pfizer, Roche. Diana Lüftner has been a consultant for Amgen, Astra Zeneca, BMS, Daiichi Sankyo, GSK, high5md, Loreal, MSD, Novartis, onkowissen.de, Pfizer, Roche, Seagen, TEVA; received honoraria from Amgen, Astra Zeneca, BMS, Daiichi Sankyo, GSK, high5md, Loreal, MSD, Novartis, onkowissen.de, Pfizer, Roche, Seagen, TEVA. Frederik Marmé received consulting fees from Astra Zeneca, Pharma, Daiichi Sankyo, EISAI, Gilead, GSK, Novartis, Myriad Genetics, Seagen, Stemline Menarini, Lilly, MSD, Pfizer, Roche, Biontech, Nerviano, and Böhringer‐Ingelheim; received honoraria /expenses from Astra Zeneca, Pharma&, Daiichi Sankyo, EISAI, Gilead, GSK, Myriad Genetics, Seagen, Stemline Menarini, Lilly, MSD, Pfizer, and Roche; received support for attending meeting and/or travel from Astra Zeneca, GSK, Gilead, Pfizer, Lilly, Stemline, Daiichi Sankyo, Roche, and Abbvie; received Advisory Board honoraria from Immutep, AMGEN, PALLEOS; have other financial or non‐financial interests with Roche (Local PI), Astra Zeneca (Coordinating PI), Novartis (Local PI), Eisai (Local PI), Gilead/Immunomedics (Coordinating PI), MSD (Local PI), Vaccibody (Local PI), GSK (Local PI), Böhringer‐Ingelheim, Pfizer/Seagen, Daiichi Sankyo, and Menarini Stemline. Marion van Mackelenbergh received personal fees, honoraria, travel grants from Amgen, Astra Zeneca, Daiichi Sankyo, Genomic Health, Gilead, GSK, Jenapharm, Lilly, Molecular Health, MSD, Mylan, Novartis, Pfizer, Pierre Fabre, Roche, Seagen. Beate Rautenberg received advisory board honoraria, speaker honoraria and travel cost reimbursement from Novartis, Astra Zeneca, Pfizer, Pierre Fabre, Roche, MSD, Lilly, Abbvie, Daiichi Sankyo; received third‐party research funding from Novartis, Daiichi Sankyo, Astra Zeneca, Lilly. Marcus Schmidt has been a consultant for Astra Zeneca, BioNTech, Eisai, Daiichi Sankyo, Eisai, GILEAD, Lilly, Molecular Health, Menarini Stemline, MSD, Novartis, Pantarhei Bioscience, Pfizer, Pierre Fabre, Roche und Seagen; has owner interests for patents EP2390370B1, EP2951317B1; received honoraria from Astra Zeneca, BioNTech, Eisai, Daiichi Sankyo, Eisai, GILEAD, Lilly, Molecular Health, Menarini Stemline, MSD, Novartis, Pantarhei Bioscience, Pfizer, Pierre Fabre, Roche und Seagen; received research funding from Astra Zeneca, BioNTech, Eisai, Genentech, German Breast Group (GBG), Novartis, Palleos, Pantarhei Bioscience, Pfizer, Pierre‐Fabre, Roche und Seagen. Pauline Wimberger received honoraria from Amgen, Astra Zeneca, Clovis, Daichii Sankyo; Eisai, Gilead, GlaxoSmithKline, Lilly, MSD, Novartis, Pfizer, Roche Pharma, TEVA, Regeneron. Christina Pfister, Claudia Quiering and Christian Roos are employees of Novartis. Achim Wöckel received consulting fees from Amgen, Astra Zeneca, Celgene, Lilly, Novartis, Pfizer, Roche, MSD, Genomic Health, Organon, Seagen, Exact Sciences, Gilead, Daiichi Sankyo, Stemline; payment or honoraria for lectures, manuscript writing or educational events from Aurikamed, Celgene, Dajiichi Sanko, Exact Sciences, Genomic Health, Gilead, Lilly, MSD, Novartis, Onkowissen, Pfizer, Roche, Seagen, Stemline. All other authors have declared no conflicts of interest.

## ETHICS STATEMENT

The study was conducted in accordance with the World Medical Association Declaration of Helsinki. The observational study protocol and informed consent (IC) forms were approved by the independent Ethics Committee of the Julius‐Maximilians University Würzburg in Germany (reference number 181/17_awb‐me). All participants provided written informed consent. This study was prospectively registered at ClinicalTrials.gov as NCT06311383.

## Supporting information


**Data S1:** Supporting Information

## Data Availability

The data that support the findings of this study are available from the corresponding authors upon reasonable request.
