# Supplementary material for: Real‐world progression‐free survival and overall survival in patients with HR +/HER2 − advanced breast cancer treated in first‐line with ribociclib, endocrine monotherapy or chemotherapy: Results from the observational RIBANNA study
Source: Int J Cancer. 2026 Mar 6;159(1):210–23. doi: 10.1002/ijc.70397 (PMC13139987; doi:10.1002/ijc.70397)
Supplement: Supplementary file 1 — Data S1: Supporting Information [file IJC-159-210-s001.pdf]

Real-world progression-free survival and overall survival in patients with HR$^+$/HER2$^-$ advanced breast cancer treated in first-line with ribociclib, endocrine monotherapy or chemotherapy: Results from the observational RIBANNA study

Peter A. Fasching, Cosima Brucker, Thomas Decker, Anne Engel, Thomas Göhler, Christian Jackisch, Jan Janssen, Andreas Köhler, Kerstin Lüdtke-Heckenkamp, Diana Lüftner, Frederik Marmé, Marion van Mackelenbergh, Beate Rautenberg, Marcus Schmidt, Rudolf Weide, Pauline Wimberger, Elena Kisseleff, Christina Pfister, Claudia Quiering, Christian Roos, Achim Wöckel

Table of Contents

**Supplementary Figures**		**Pages 2–5**
Supplement-Figure S 1	RIBANNA study design	Page 2
Supplement-Figure S 2	Cox proportional hazard analysis of selected adverse prognostic factors for progression-free survival in first-line therapy in the ribociclib cohort	Pages 3–4
Supplement-Figure S 3	Progression-free survival-2 in the ribociclib cohort (FAS)	Page 5
**Supplementary Tables**		**Pages 6–16**
Supplement-Table S 1	Full set of evaluation criteria in the RIBANNA study	Page 6
Supplement-Table S 2	Type of second-line therapy depending on the first-line therapy among patients with at least one second therapy (FAS)	Page 7
Supplement-Table S 3	Progression-free survival on first-line treatment in the ET and CT cohorts at cutoff date – unadjusted data (FAS)	Page 8
Supplement-Table S 4	Overview of median PFS and median OS observed in the placebo-controlled, pivotal Phase 3 trials MONALEESA-2, -3, and -7	Page 9
Supplement-Table S 5	Post hoc definition of ML-like subgroups within the ribociclib cohort (FAS)	Page 10
Supplement-Table S 6	Progression-free survival on first-line treatment in the ribociclib cohort separated by attributability of patients to one of the three pivotal MONALEESA trials (FAS) or non-attributable patients to either of the trials	Page 11
Supplement-Table S 7	Progression-free survival-2 (PFS2) in the ribociclib cohort (FAS)	Page 11
Supplement-Table S 8	Occurrence of adverse events of interest on first-line treatment by maximum severity in the ribociclib cohort (SAF)	Pages 12–14
Supplement-Table S 9	Reasons for discontinuation of therapy in the ribociclib cohort in first line (SAF)	Page 14
Supplement-Table S 10	Selected demographic and other baseline characteristics in the ML-like subgroups within the ribociclib cohort (FAS)	Pages 15–16

SUPPLMENTARY FIGURES

Supplement-Figure S 1: RIBANNA study design

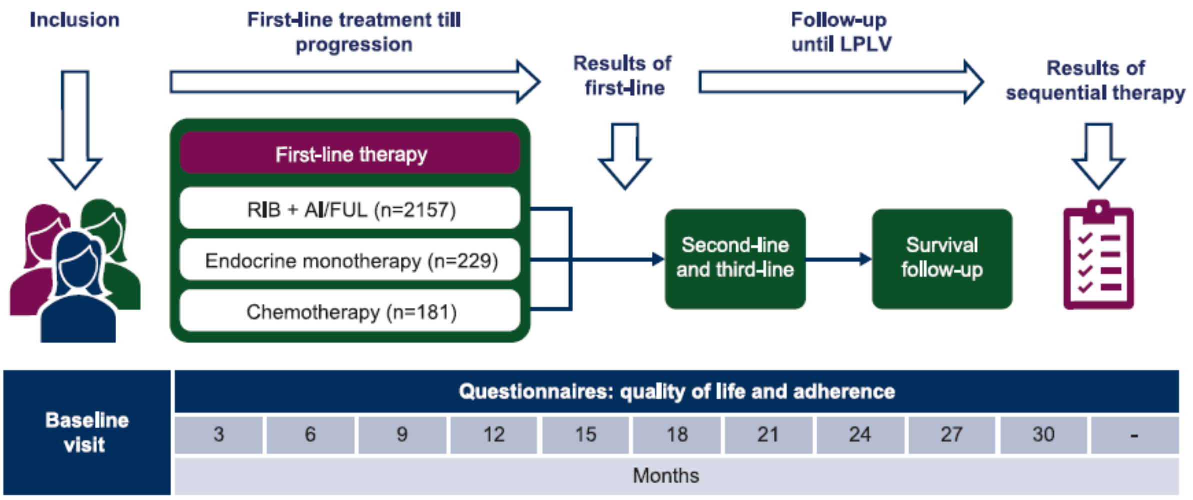

AI=Aromatase inhibitor; FUL=Fulvestrant; LPLV=last patient's last visit

Supplement-Figure S 2: Cox proportional hazard analysis of selected adverse prognostic factors for progression-free survival in first-line therapy in the ribociclib cohort

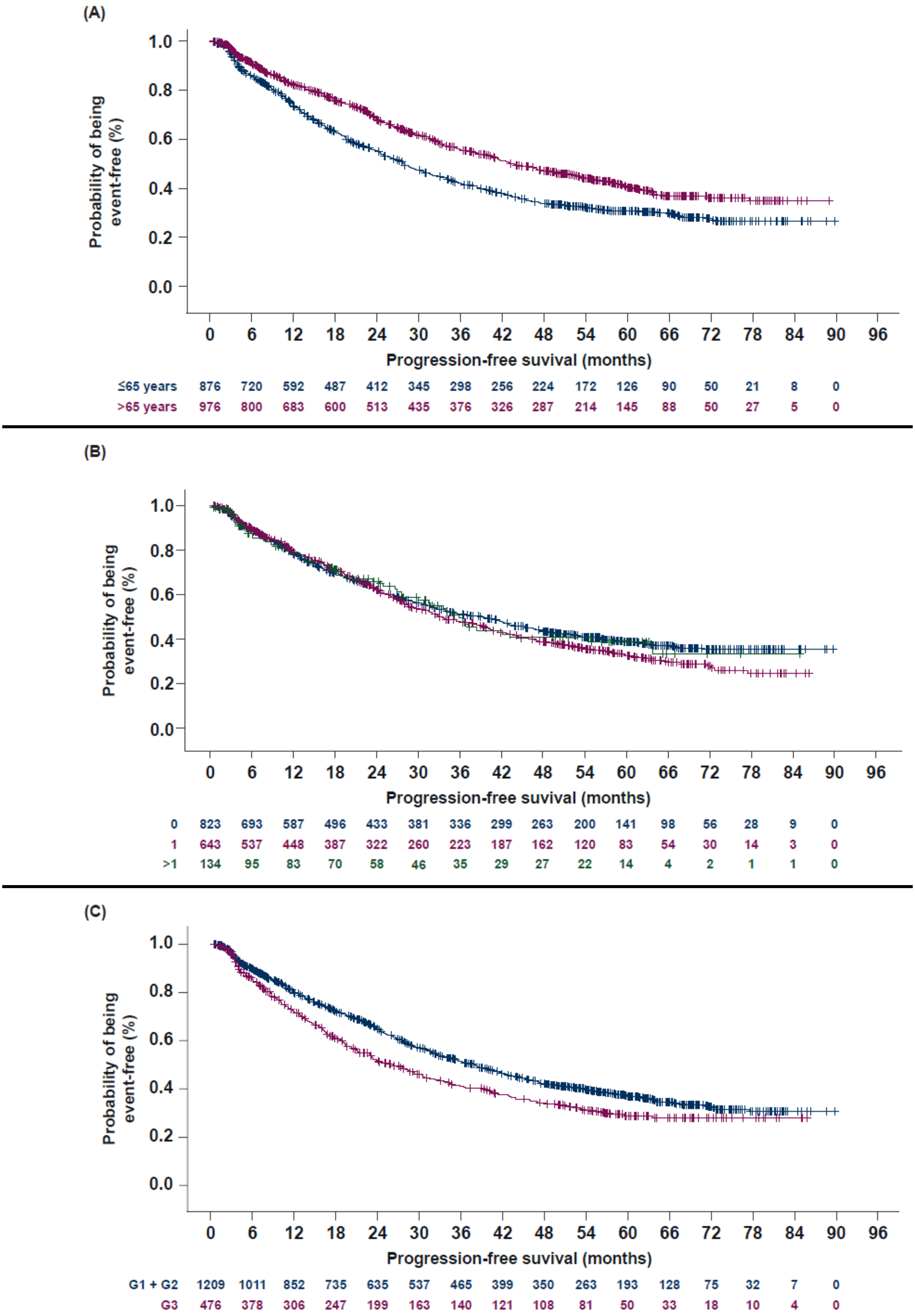

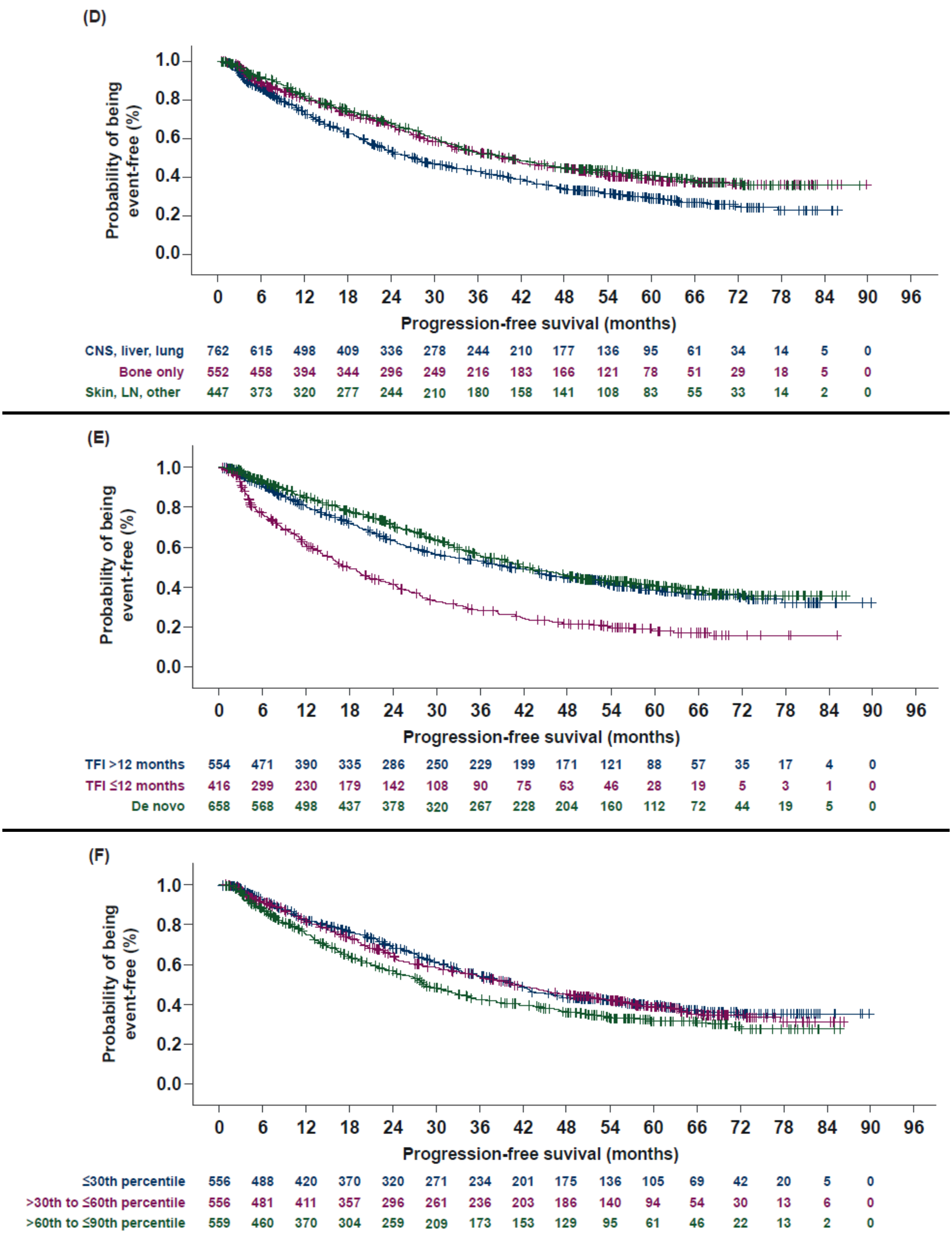

(A) Progression-free survival according to age (≤65 years vs. >65 years).
(B) Progression-free survival according to ECOG-PS (0 vs. 1 vs. >1).
(C) Progression-free survival according to grading at initial diagnosis (G1+G2 vs. G3).
(D) Progression-free survival according to metastasis site (CNS/liver/lung vs. bone only vs. skin/lymph nodes/other).
(E) Progression-free survival according to treatment-free interval (>12 months vs. ≤12 months vs. de novo).
(F) Progression-free survival according to ribociclib relative dose intensity (≤30th percentile [1.96–69.76% relative DI] vs. >30th to ≤60th percentile [70–99.91% relative DI] vs. >60th to ≤90th percentile [100–104.76% relative DI]).
AI=Aromatase inhibitor; CNS=Central nervous system; DI=Dose intensity; ECOG-PS= Eastern Cooperative Oncology Group performance status; F=Fulvestrant; G=grade; LN=lymph nodes; TFI: Treatment-free interval.

Supplement-Figure S 3: Progression-free survival-2 in the ribociclib cohort (FAS)

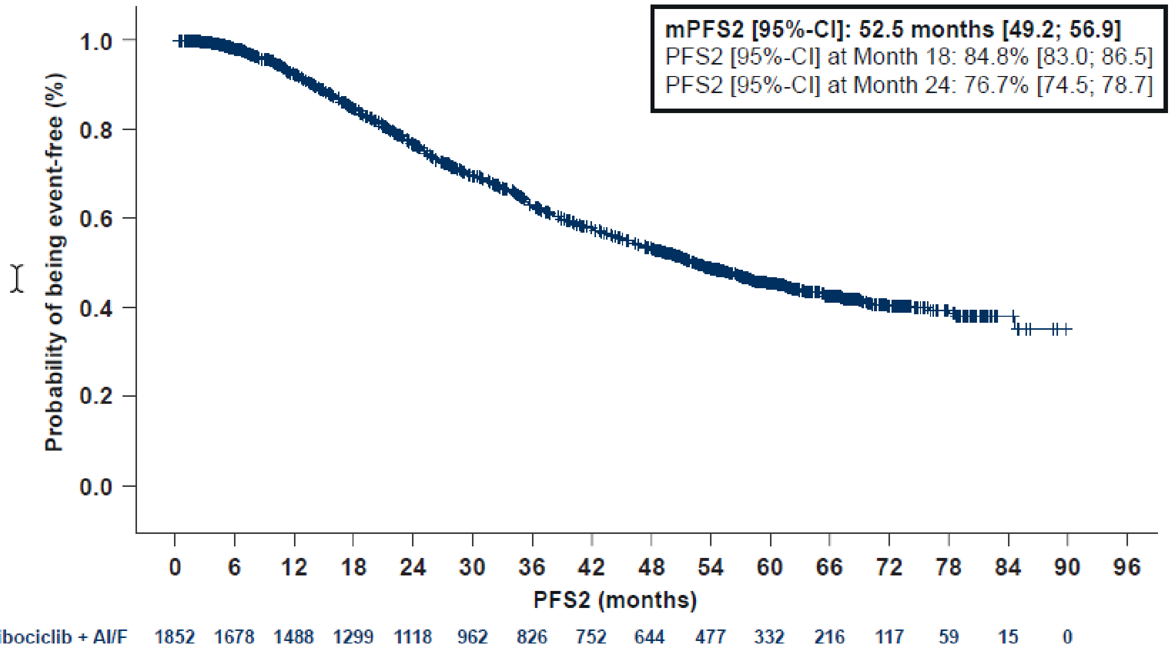

mPFS2=Median progression-free survival in second line counted from treatment start onwards.

SUPPLEMENTARY TABLES

Supplement-Table S 1: Full set of evaluation criteria in the RIBANNA study

**Clinical**	Median progression-free survival (PFS) in first line, 2nd line, and 3rd line (time from first treatment in respective line until progression or death from any cause)<br>Second progression-free survival (PFS2; time from first treatment in 2nd line until second progression or death from any cause, all patients)<br><br>Conditional PFS (cPFS) for progression-free patients at Months 12, 24, and 36<br>Overall survival (OS)<br>Time to treatment failure (TTF)<br>Time to first chemotherapy<br>Time to next treatment/therapy (TTNT)
**Imaging**	Clinical and radiological time point assessments (RECIST 1.1)<br>(as far as available from routine care)
**Quality of life / PRO**	Morisky Medication Adherence Scale with 8 items (MMAS-8; only for patients in the ribociclib cohort during first line)<br>EORTC Quality of Life Questionnaire (QLQ) C30<br>EORTC QLQ BR23<br>Hospital Anxiety and Depression Scale – German Version (HADS-D)
**Safety**	Treatment-emergent adverse events, vital signs, proportions of patients with dose reduction, dose interruption, or permanent discontinuation

EORTC=European Organization for Research and Treatment of Cancer; HADS=Hospital Anxiety and Depression Scale; MMAS=Morisky Medication Adherence Scale; OS=Overall survival; PFS=Progression-free survival; PRO=Patient-reported outcome; QLQ=Quality of Life Questionnaire; RECIST=Response Evaluation Criteria in Solid Tumors; TTF=Time to treatment failure; TTNT=Time to next treatment/therapy

Supplement-Table S 2: Type of second-line therapy depending on the first-line therapy among patients with at least one second therapy (FAS)

	**Ribociclib + AI/FUL** ***(N=850)*** **n (%)**	**Endocrine monotherapy** ***(N=82)*** **n (%)**	**Chemotherapy** ***(N=67)*** **n (%)**	**Total** ***(N=999)*** **n (%)**
Abemaciclib*	18 (2.1)	1 (1.2)	1 (1.5)	20 (2.0)
Everolimus	71 (8.4)	2 (2.4)	1 (1.5)	74 (7.4)
CDK 4/6 inhibitor	8 (0.9)	0 (0.0)	0 (0.0)	8 (0.8)
Chemotherapy	303 (35.6)	13 (15.9)	33 (49.3)	349 (34.9)
Endocrine monotherapy	227 (26.7)	33 (40.2)	17 (25.4)	277 (27.7)
Palbociclib*	16 (1.9)	12 (14.6)	4 (6.0)	32 (3.2)
Ribociclib*	132 (15.5)	20 (24.4)	7 (10.4)	159 (15.9)
Other	75 (8.8)	1 (1.2)	4 (6.0)	80 (8.0)

AI=Aromatase inhibitor; FAS=Full analysis set; FUL=Fulvestrant; * in combination with endocrine therapy according to the respective summaries of product characteristics (SmPCs)

Supplement-Table S 3: Progression-free survival on first-line treatment in the ET and CT cohorts at cutoff date – unadjusted data (FAS)

	**Endocrine therapy (ET)** ***(N=183)***	**Chemotherapy (CT)** ***(N=139)***
No. of censored observations	82	55
No. of events	101	84
KM estimator for time to event (months)		
75%-Quantile [95%-CI]	11.8 [7.6; 18.0]	6.1 (4.6, 8.8)
50%-Quantile [95%-CI]	37.4 [26.6; 45.5]	17.0 [13.5; 20.5]
25%-Quantile [95%-CI]	84.6 [56.5, NR]	39.7 [25.4; NR]
KM estimator for time to event (%) at:		
Month 18 after reference time point [95%-CI]	68.6 [60.9; 75.1]	46.4 [36.7; 55.5]
Month 24 after reference time point [95%-CI]	62.3 [54.3; 69.4]	34.3 [25.1; 43.6]

CI=Confidence interval; FAS=Full analysis set; KM=Kaplan-Meier; NR=Not reached

Supplement-Table S 4: Overview of median PFS and median OS observed in the placebo-controlled, pivotal Phase 3 trials MONALEESA-2, -3, and -7

	MONALEESA-2	MONALEESA-3	MONALEESA-7
**Trial Indication**	aBC in postmenopausal women		aBC in pre-menopausal women
**Treatment line**	1L (no prior treatment for advanced disease)	1L (no prior treatment for advanced disease) **or** 2L (i.e., one prior line of ET)	1L (no prior treatment for advanced disease) **or** 2L (i.e., one prior line of CT; administered to only 14% of patients in each arm)
**Trial treatment**	Ribociclib + letrozole vs. Placebo + letrozole	Ribociclib + fulvestrant vs. Placebo + fulvestrant	Ribociclib + TAM or NSAI + goserelin vs. Placebo + TAM or NSAI + goserelin
**No. of patients**	Ribociclib arm ITT: N=334<br>Placebo arm ITT: N=334	Ribociclib arm ITT: N=484 (1L: 237)<br>Placebo arm ITT: N=242 (1L: 128)	Ribociclib arm ITT: N=335<br>Placebo arm ITT: N=337<br>Patients with AI: N=495 (Ribociclib+AI: 248; Placebo+AI: 247)
**Primary analysis of PFS (investigator-reported)**<br>mPFS [95%-CI]<br>Hazard ratio [95%-CI]	Median duration of FU: 26.4 months [21]<br>Ribociclib: **25.3 months** [23.0; 30.3]<br>Placebo: 16.0 months [13.4; 18.2]<br>HR=0.568 [0.457; 0.704]; *$p=9.63x10^{-8}$* *	Median duration of FU: 20.4 months [17]<br>Ribociclib: 20.5 months [18.5; 23.5]<br>Placebo: 12.8 months [10.9; 16.3]<br>HR=0.593 [0.480; 0.732]; *$p<0.001$*	Median duration of FU: 19.2 months [19]<br>Ribociclib: **23.8 months** [19.2; NR]<br>Placebo: 13.0 months [11.0; 16.4]<br>HR=0.55 [0.44; 0.69]; *$p<0.0001$*
**Analysis of OS**<br>mPFS [95%-CI]<br>Hazard ratio [95%-CI]	Median duration of FU: 80 months [20]<br>Ribociclib: **63.9 months** [52.4; 71.0]<br>Placebo: 51.4 months [47.2; 59.7]<br>HR=0.76 [0.63; 0.93]; *$p=0.008$*	Median duration of FU: 56.3 months [24]<br>Ribociclib: 53.7 months [46.9, NR]<br>Placebo: 41.5 months [37.4; 49.0]<br>HR=0.73 [0.59; 0.90]	Median duration of FU: 53.5 months [25]<br>Ribociclib: **58.7 months** (no CI reported)<br>Placebo: 48.0 months [37.8; NR]<br>HR=0.76 [0.61; 0.96]
**Additional information**	* Formally, the primary endpoint was already met at the first interim analysis after a median FU of 15.3 months, when the HR for progression-free survival was HR=0.56; 95%-CI:0.43–0.72; *$p=3.29 \times 10^{-6}$* [15]. The mPFS for ribociclib was not reached at that time.	<u>Results in first line</u><br>- PFS (median FU: 39.4 months) [23]<br>mPFS Ribociclib: **33.6 months** [27.1; 41.3]<br>mPFS Placebo: 19.2 months [14.9; 23.6]<br>HR=0.55 [0.42; 0.72]<br>- OS (median FU 70.8 months) [22]<br>mOS Ribociclib: **67.6 months** [59.6; NR]<br>mOS Placebo: 51.8 months [40.4; 57.6]<br>HR=0.67; [0.50; 0.90]	

1L=First line; 2L=Second line; aBC=Advanced breast cancer; AI=Aromatase inhibitor; CI=Confidence interval; ET=Endocrine therapy; FU=Follow-Up; HR=Hazard ratio; ITT=Intent-to-treat (population); mPFS=Median progression-free survival; mOS=Median overall survival; NR=Not reached; NSAI=Non-steroidal aromatase inhibitor; TAM=Tamoxifen

Supplement-Table S 5: *Post hoc* definition of ML-like subgroups within the ribociclib cohort (FAS)

**ML2-like subgroup**<br>***(N=560)***	**ML3-like subgroup**<br>***(N=203)***	**ML7-like subgroup**<br>***(N=59)***
**Inclusion criteria**<br>- Postmenopausal patients with 1L RIB + LET<br>- ECOG PS 0 or 1 at baseline<br>- ≥12 months of therapy-free interval, if ET (NSAI, LET, or ANA as prior antineoplastic therapy)	**Inclusion criteria**<br>- Postmenopausal patients with 1L RIB + FUL<br>- ECOG PS 0 or 1 at baseline	**Inclusion criteria**<br>- Premenopausal or perimenopausal patients aged <60 years, with 1L RIB + LET or ANA (and ≥1 intake of GNRH/LNHR-analog)<br>- ECOG PS 0 or 1 at baseline<br>- Only 1L *de novo* patients, or patients with ≥12 months therapy-free interval between prior antineoplastic therapy with NSAI (LET or ANA) and study start, prior therapy with SERD or tamoxifen are not subject to any restriction
**Exclusion criteria [a]**<br>- Angina pectoris, pericarditis, myocardial infarction within 12 months prior to study start<br>- Cardiac arrhythmias, e.g., ventricular, supraventricular, nodal arrhythmias in the previous 12 months	**Exclusion criteria [a]**<br>- SERD like FUL in prior antineoplastic therapy<br>- Warfarin or coumarin at time of informed consent<br>- Angina pectoris, pericarditis, myocardial infarction, bypass, CABG within 6 months prior to study start	**Exclusion criteria [a]**<br>- Cardiac arrhythmias, e.g., ventricular supraventricular, nodal arrhythmias in previous 12 months<br>- Warfarin or coumarin at the time of informed consent

1L=First line; ANA=Anastrozole; CABG=Coronary artery bypass graft; FUL=Fulvestrant; GNRH/LNHR=Gonadotropin releasing hormone; LET=Letrozole; ML=MONALEESA (trial); NSAI=Non-steroidal aromatase inhibitor; PS=Performance status; RIB=Ribociclib; SERD=Selective estrogen receptor degrader

a: Other common exclusion criteria were central nervous system metastases at start of study, pulse <50 bpm or >90 bpm at baseline, QTcF >450 msec at baseline (QT interval corrected using the Fridericia formula), systolic blood pressure at baseline >160 mmHg or <90 mmHg, any surgery <14 days before study start, radiotherapy ≤4 weeks before study start, HIV infection, and cardiomyopathy.

Supplement-Table S 6: Progression-free survival on first-line treatment in the ribociclib cohort separated by attributability of patients to one of the three pivotal MONALEESA trials (FAS) or non-attributable patients to either of the trials

	**Ribociclib + AI/FUL cohort *(N=1852)***			
	**ML2-like subgroup *(N=560)***	**ML3-like subgroup *(N=203)***	**ML7-like subgroup *(N=59)***	**Non-attributable *(N=1030)***
No. of censored observations	279	74	24	476
No. of events	281	129	35	554
KM estimator for time to event (months)				
75%-Quantile [95%-CI]	17.9 [14.3; 21.7]	9.9 [7.2; 13.8]	10.3 [4.2; 14.2]	14.0 [12.0; 16.4]
50%-Quantile [95%-CI]	43.9 [36.4; 49.5]	22.9 [18.0; 25.8]	30.3 [16.1; NR]	35.5 [31.7; 38.6]
25%-Quantile [95%-CI]	NR	66.0 [42.8; NR]	NR	NR
KM estimator for time to event (%) at:				
Month 18 after reference time point [95%-CI]	74.7 [70.7; 78.3]	57.3 [49.8; 64.2]	60.4 [46.7; 71.7]	69.6 [66.5; 72.5]
Month 24 after reference time point [95%-CI]	66.7 [62.3; 70.6]	47.1 [39.6; 54.3]	53.3 [39.7; 65.2]	62.4 [59.2; 65.5]

AI=Aromatase inhibitor; CI=Confidence interval; FAS=Full analysis set; FUL=Fulvestrant; KM=Kaplan-Meier; ML=MONALEESA (trial); NR=Not reached

Note: The attribution of patients to one of the three pivotal trials was done based on obvious similarities of the in-exclusion criteria as documented in the respective full-paper publications. Thus, the attribution to the category “other” does not necessarily imply incompatibility with one of the three pivotal trials, because missing data might have precluded a proper attribution. The p-value for global differences across subgroups derived from log-rank test was $p=0.0010$.

Supplement-Table S 7: Progression-free survival-2 (PFS2) in the ribociclib cohort (FAS)

	**Ribociclib + AI/FUL (*N=1852)***
No. of censored observations	1067
No. of events	785
KM estimator for time to event (months)	
75%-Quantile [95%-CI	25.3 [23.8; 26.9]
50%-Quantile [95%-CI]	52.5 [49.2; 56.9]
25%-Quantile [95%-CI]	NR
KM estimator for time to event (%) at:	
Month 18 after reference time point [95%-CI]	84.8 [83.0; 86.5]
Month 24 after reference time point [95%-CI]	76.7 [74.5; 78.7]

AI=Aromatase inhibitor; CI=Confidence interval; FAS=Full analysis set; FUL=Fulvestrant; KM=Kaplan-Meier; NR=Not reached

PFS2: Time from first intake of the respective medication in first line up to progression or death after start of second-line treatment.

Supplement-Table S 8: Occurrence of adverse events of interest on first-line treatment by maximum severity in the ribociclib cohort (SAF)

	**Ribociclib + AI/FUL** ***(N=1999)*** **n (%)**
**Any adverse event (not limited to AESIs)**	**1849 (92.5)**
Missing grade	2 (0.1)
Grade 1	142 (7.1)
Grade 2	591 (29.6)
Grade 3	768 (38.4)
Grade 4	159 (8.0)
Grade 5	187 (9.4)
Grade ≥3	***1114 (55.7)***
HEPATOBILIARY TOXICITY	
**Alanine aminotransferase (ALT) increased**	**83 (4.2)**
Missing grade	2 (0.1)
Grade 1	9 (0.5)
Grade 2	26 (1.3)
Grade 3	39 (2.0)
Grade 4	7 (0.4)
Grade ≥3	***46 (2.3)***
**Aspartate aminotransferase (AST) increased**	**80 (4.0)**
Missing grade	3 (0.2)
Grade 1	18 (0.9)
Grade 2	22 (1.1)
Grade 3	34 (1.7)
Grade 4	3 (0.2)
Grade ≥3	***37 (1.9)***
**Blood bilirubin increased**	**13 (0.7)**
Grade 1	4 (0.2)
Grade 2	5 (0.3)
Grade 3	4 (0.2)
Grade ≥3	***4 (0.2)***
**Hyperbilirubinemia**	**2 (0.1)**
Grade 2	1 (0.1)
Grade 4	1 (0.1)
Grade ≥3	***1 (0.1)***
INFECTIOINS AND INFESTATIONS	
**COVID-19**	**126 (6.3)**
Grade 1	56 (2.8)
Grade 2	60 (3.0)
Grade 3	7 (0.4)
Grade 4	1 (0.1)
Grade 5	2 (0.1)
Grade ≥3	***10 (0.5)***
**Nasopharyngitis**	**127 (6.4)**
Grade 1	78 (3.9)
Grade 2	47 (2.4)
Grade 3	2 (0.1)
Grade ≥3	***2 (0.1)***
**Urinary tract infection**	**108 (5.4)**
Missing grade	1 (0.1)

Grade 1	30 (1.5)
Grade 2	66 (3.3)
Grade 3	10 (0.5)
Grade 5	1 (0.1)
Grade ≥3	***11 (0.6)***
MYELOSUPPRESSION	
**Anemia**	**200 (10.0)**
Grade 1	48 (2.4)
Grade 2	96 (4.8)
Grade 3	54 (2.7)
Grade 4	2 (0.1)
Grade ≥3	***56 (2.8)***
**Leukopenia**	**434 (21.7)**
Grade 1	80 (4.0)
Grade 2	226 (11.3)
Grade 3	119 (6.0)
Grade 4	9 (0.5)
Grade ≥3	***128 (6.4)***
**Neutropenia**	**541 (27.1)**
Missing grade	2 (0.1)
Grade 1	48 (2.4)
Grade 2	151 (7.6)
Grade 3	313 (15.7)
Grade 4	26 (1.3)
Grade 5	1 (0.1)
Grade ≥3	***340 (17.0)***
**Thrombocytopenia**	**120 (6.0)**
Missing grade	1 (0.1)
Grade 1	61 (3.1)
Grade 2	33 (1.7)
Grade 3	20 (1.0)
Grade 4	4 (0.2)
Grade 5	1 (0.1)
Grade ≥3	***25 (1.3)***
RENAL AND URINARY DISORDERS	
**Acute kidney injury**	**25 (1.3)**
Missing grade	1 (0.1)
Grade 2	5 (0.3)
Grade 3	16 (0.8)
Grade 4	2 (0.1)
Grade 5	1 (0.1)
Grade ≥3	***19 (1.0)***
**Renal failure**	**38 (1.9)**
Grade 1	5 (0.3)
Grade 2	15 (0.8)
Grade 3	9 (0.5)
Grade 4	6 (0.3)
Grade 5	3 (0.2)
Grade ≥3	***18 (0.9)***
**Urinary retention**	**21 (1.1)**
Grade 1	5 (0.3)
Grade 2	9 (0.5)

Grade 3	7 (0.4)
Grade ≥3	***3 (0.4)***
**CARDIAC DISORDERS**	
**Electrocardiogram QT interval prolonged**	**78 (3.9)**
Missing grade	2 (0.1)
Grade 1	28 (1.4)
Grade 2	33 (1.7)
Grade 3	15 (0.8)
Grade ≥3	***15 (0.8)***
**SKIN AND SUBCUTANEOUS TISSUE DISORDERS**	
**Vitiligo**	**6 (0.3)**
Grade 1	2 (0.1)
Grade 2	4 (0.2)
Grade ≥3	***0 (0.0)***

AESI=Adverse event of interest; AI=Aromatase inhibitor; FUL=Fulvestrant; SAF=Safety analysis set

Supplement-Table S 9: Reasons for discontinuation of therapy in the ribociclib cohort in first line (SAF)

	**Ribociclib + AI/FUL cohort** ***(N=1999)***
**Any reason**	**1218 (60.9)**
Adverse event	24 (1.2)
Death	184 (9.2)
Withdrawal of informed consent	5 (0.3)
Disease progression	918 (45.9)
Lost to Follow-Up	16 (0.8)
Physician decision	28 (1.4)
Patient decision	37 (1.9)
Other reason	14 (0.7)

AI=Aromatase inhibitor; FUL=Fulvestrant; SAF=Safety analysis set

Note: Data as documented on therapy eCRF as “Reason for end of therapy”. Data are based on number of patients; multiple specifications per patient were possible.

Supplement-Table S 10: Selected demographic and other baseline characteristics in the ML-like subgroups within the ribociclib cohort (FAS)

		Ribociclib + AI/FUL cohort *(N=1852)*				
		**ML2-like subgroup *(N=560)***	**ML3-like subgroup *(N=203)***	**ML7-like subgroup *(N=59)***	**Other [a, b] *(N=1030)***	***P-value* [c]**
Age (years)	Mean ± SD	67.0 ± 10.3	67.0 ± 9.1	45.7 ± 5.0	65.6 ± 11.9	*<0.001*
	Median	68.0	66.0	46.0	66.0	
	Range	36.0-90.0	38.0-88.0	34.0-56.0	27.0-92.0	
Age groups, n (%)	<75 years	386 (68.9)	156 (76.8)	59 (100.0)	755 (73.3)	*<0.001*
	75-≤80 years	129 (23.0)	34 (16.7)	0 (0.0)	178 (17.3)	
	>80 years	45 (8.0)	13 (6.4)	0 (0.0)	97 (9.4)	
Menopausal status, n (%)	Pre-menopausal	0 (0.0)	0 (0.0)	47 (79.7)	95 (9.2)	*<0.001*
	Peri-menopausal	0 (0.0)	0 (0.0)	12 (20.3)	39 (3.8)	
	Post-menopausal	560 (100.0)	203 (100.0)	0 (0.0)	879 (85.3)	
	Missing data	0 (0.0)	0 (0.0)	0 (0.0)	17 (1.7)	
Baseline EOCG PS, n (%)	0	329 (58.8)	103 (50.7)	50 (84.7)	341 (33.1)	*<0.001*
	1	231 (41.3)	100 (49.3)	9 (15.3)	303 (29.4)	
	>1	0 (0.0)	0 (0.0)	0 (0.0)	134 (13.0)	
	Missing data	0 (0.0)	0 (0.0)	0 (0.0)	252 (24.5)	
Grading, n (%)	G1+G2	366 (65.4)	135 (66.5)	34 (57.6)	674 (65.4)	*0.200*
	G3	151 (27.0)	56 (27.6)	22 (37.3)	247 (24.0)	
	Missing data	43 (7.7)	12 (5.9)	3 (5.1)	109 (10.6)	
Metastases at study start, n (%)	CNS, liver, lung	219 (39.1)	83 (40.9)	15 (25.4)	445 (43.2)	*0.006*
	Bone only	145 (25.9)	60 (29.6)	25 (42.4)	322 (31.3)	
	Skin, lymph nodes, other	157 (28.0)	53 (26.1)	14 (23.7)	223 (21.7)	
	Missing data	39 (7.0)	7 (3.4)	5 (8.5)	40 (3.9)	
Treatment-free interval, n (%)	Missing information for performed prior antineoplastic therapy	3 (0.5)	1 (0.5)	0 (0.0)	17 (1.7)	*<0.001*
	TFI >12 months	167 (29.8)	79 (38.9)	9 (15.3)	299 (29.0)	
	TFI ≤12 months	69 (12.3)	85 (41.9)	18 (30.5)	244 (23.7)	
	De novo [d]	265 (47.3)	18 (8.9)	27 (45.8)	348 (33.8)	
	Missing data	56 (10.0)	20 (9.9)	5 (8.5)	122 (11.8)	
Any prior chemotherapy, n (%)	No	377 (67.3)	91 (44.8)	35 (59.3)	657 (63.8)	*<0.001*
	Yes	183 (32.7)	112 (55.2)	24 (40.7)	373 (36.2)	

Previous antineoplastic therapy	Endocrine therapy	55 (9.8)	44 (21.7)	4 (6.8)	166 (16.1)	
	Chemotherapy	103 (18.4)	44 (21.7)	12 (20.3)	147 (14.3)	
	Endocrine therapy + Chemotherapy	80 (14.3)	68 (33.5)	12 (20.3)	226 (21.9)	*<0.001*
	No previous therapy	322 (57.5)	47 (23.2)	31 (52.5)	491 (47.7)	

AI=Aromatase inhibitor; CNS=Central nervous system; FAS=Full analysis set; FUL=Fulvestrant; MONALEESA (trial); PS=Performance status; SD=Standard deviation; TFI=Treatment-free interval

a: The category “other” captures those cases that could not be clearly attributed to one of the three ML-like subgroups for any reason, including lack of required information. Thus, this subgroup might also include patients who would have met the criteria for one of the three ML trials but could not be attributed due to missing data.

b: In this subgroup, only 31 patients who had received fulvestrant in combination with ribociclib were pre- or perimenopausal. Thus, the label extension (which was not investigated in MONALEESA-7) was not responsible for the large number on non-attributable patients.

c: Global p-values derived from F-Test (continuous data) or Chi-square test (categorical data) for inhomogeneity (differences) across the three cohorts.

d: Patients who did not receive any prior antineoplastic treatment.
